# The Role of Social Deprivation and Cannabis Use in Explaining Variation in the Incidence of Psychotic Disorders: Findings From the EU-GEI Study

**DOI:** 10.1093/schbul/sbae072

**Published:** 2024-05-24

**Authors:** Vera Brink, Humma Andleeb, Charlotte Gayer-Anderson, Celso Arango, Manuel Arrojo, Domenico Berardi, Miquel Bernardo, Julio Bobes, Cristina Marta Del-Ben, Laura Ferraro, Lieuwe de Haan, Daniele La Barbera, Caterina La Cascia, Antonio Lasalvia, Pierre-Michel Llorca, Paolo Rossi Menezes, Baptiste Pignon, Julio Sanjuán, José Luis Santos, Jean-Paul Selten, Ilaria Tarricone, Andrea Tortelli, Giada Tripoli, Eva Velthorst, Bart P F Rutten, Jim van Os, Diego Quattrone, Robin M Murray, Peter B Jones, Craig Morgan, Marta Di Forti, Hannah E Jongsma, James B Kirkbride

**Affiliations:** Department of Psychosis, University Center Psychiatry, University Medical Center Groningen, University of Groningen, Groningen, The Netherlands; PsyLife Group, Division of Psychiatry, University College London, London, UK; PsyLife Group, Division of Psychiatry, University College London, London, UK; ESRC Centre for Society and Mental Health, King’s College London, London, UK; Department of Health Service and Population Research, Institute of Psychiatry, Psychology and Neuroscience, King’s College London, London, UK; Department of Child and Adolescent Psychiatry, Institute of Psychiatry and Mental Health, Hospital General Universitario Gregorio Marañón, School of Medicine, Universidad Complutense, IiSGM, CIBERSAM, Madrid, Spain; Department of Mental Health and Drug-Addiction Assistance, Health Service of Galicia, Psychiatric Genetic Group IDIS, Hospital Clínico Universitario de Santiago de Compostela, affiliated center to Centro de Investigación Biomédica en Red de Salud Mental, Servicio Gallego de Salud, Santiago de Compostela, Spain; Alma Mater Studiorium Università di Bologna, Bologna, Italy; Barcelona Clinic Schizophrenia Unit, Hospital Clinic de Barcelona, Barcelona, Spain; Departament de Medicina, Institut de Neurociències (UBNeuro), Universitat de Barcelona (UB), Barcelona, Spain; Institut d’Investigacions Biomèdiques August Pi I Sunyer (IDIBAPS), Barcelona, Spain; CIBERSAM, ISCIII, Barcelona, Spain; Department of Medicine, Psychiatry Area, School of Medicine, Universidad de Oviedo, Centro de Investigación Biomédica en Red de Salud Mental, Oviedo, Spain; Department of Neuroscience and Behavior, Ribeirão Preto Medical School, University of São Paulo, São Paulo, Brazil; Department of Biomedicine, Neuroscience, and Advanced Diagnostics, Section of Psychiatry, University of Palermo, Palermo, Italy; Department of Psychiatry, Early Psychosis Section, Academic Medical Centre, University of Amsterdam, Amsterdam, The Netherlands; Department of Biomedicine, Neuroscience, and Advanced Diagnostics, Section of Psychiatry, University of Palermo, Palermo, Italy; Department of Biomedicine, Neuroscience, and Advanced Diagnostics, Section of Psychiatry, University of Palermo, Palermo, Italy; Department of Neuroscience, Biomedicine and Movement, Section of Psychiatry, University of Verona, Verona, Italy; Fondation FondaMental, Créteil, France; CMP B CHU, Clermont-Ferrand, France; Université Clermont Auvergne, Clermont-Ferrand, France; Department of Preventive Medicine, Faculdade de Medicina, Universidade de São Paulo, São Paulo, Brazil; Núcleo de Pesquina em Saúde Mental Populacional, Universidade de São Paulo, São Paulo, Brazil; Fondation FondaMental, Créteil, France; Université Paris-Est-Créteil (UPEC) and AP-HP, Hôpitaux Universitaires « H. Mondor », DMU IMPACT, Psychiatry department and INSERM, IMRB, Translational Neuropsychiatry, Créteil, France; Department of Psychiatry, School of Medicine, Universidad de Valencia, Centro de Investigación Biomédica en Red de Salud Mental, Valencia, Spain; Department of Psychiatry, Servicio de Psiquiatría Hospital “Virgen de la Luz”, Cuenca, Spain; Department of Psychiatry and Neuropsychology, School for Mental Health and Neuroscience, Maastricht University Medical Centre, Maastricht, The Netherlands; Department of Medical and Surgical Science, Psychiatry Unit, Alma Mater Studiorium Università di Bologna, Bologna, Italy; Institut National de la Santé et de la Recherche Médicale, U955, Créteil, France; Pôle Psychiatrie Précarité, Groupe Hospitalier Paris Psychiatrie Neurosciences, Paris, France; Department of Biomedicine, Neuroscience, and Advanced Diagnostics, Section of Psychiatry, University of Palermo, Palermo, Italy; Department of Psychosis Studies, Institute of Psychiatry, Psychology and Neuroscience, King’s College London, London, UK; Department of Research, Mental Health Organization “GGZ Noord-Holland-Noord”, Heerhugowaard, The Netherlands; Department of Psychiatry and Neuropsychology, School for Mental Health and Neuroscience, Maastricht University Medical Centre, Maastricht, The Netherlands; Department of Psychiatry and Neuropsychology, School for Mental Health and Neuroscience, Maastricht University Medical Centre, Maastricht, The Netherlands; Department of Psychosis Studies, Institute of Psychiatry, Psychology and Neuroscience, King’s College London, London, UK; Brain Center, University Medical Center Utrecht, Utrecht, The Netherlands; Department of Biomedicine, Neuroscience, and Advanced Diagnostics, Section of Psychiatry, University of Palermo, Palermo, Italy; National Institute for Health Research (NIHR) Mental Health Biomedical Research Centre at South London and Maudsley NHS Foundation Trust and King’s College London, London, UK; Social, Genetic and Developmental Psychiatry Centre, Institute of Psychiatry, Psychology and Neuroscience, King’s College London, London, UK; Department of Psychosis Studies, Institute of Psychiatry, Psychology and Neuroscience, King’s College London, London, UK; National Institute for Health Research (NIHR) Mental Health Biomedical Research Centre at South London and Maudsley NHS Foundation Trust and King’s College London, London, UK; Department of Psychiatry, University of Cambridge, Herchel Smith Building for Brain & Mind Sciences, Cambridge, UK; CAMEO Early Intervention Service, Cambridgeshire and Peterborough National Health Service Foundation Trust, Chesterton Medical Centre, Cambridge, UK; ESRC Centre for Society and Mental Health, King’s College London, London, UK; Department of Health Service and Population Research, Institute of Psychiatry, Psychology and Neuroscience, King’s College London, London, UK; National Institute for Health Research (NIHR) Mental Health Biomedical Research Centre at South London and Maudsley NHS Foundation Trust and King’s College London, London, UK; Social, Genetic and Developmental Psychiatry Centre, Institute of Psychiatry, Psychology and Neuroscience, King’s College London, London, UK; Department of Psychosis, University Center Psychiatry, University Medical Center Groningen, University of Groningen, Groningen, The Netherlands; Veldzicht Centre for Transcultural Psychiatry, Balkbrug, The Netherlands; PsyLife Group, Division of Psychiatry, University College London, London, UK; Department of Psychiatry, University of Cambridge, Herchel Smith Building for Brain & Mind Sciences, Cambridge, UK

**Keywords:** social determinants of health, epidemiology, substance use, social inequality, etiology

## Abstract

**Background and Hypothesis:**

Recent findings suggest the incidence of first-episode psychotic disorders (FEP) varies according to setting-level deprivation and cannabis use, but these factors have not been investigated together. We hypothesized deprivation would be more strongly associated with variation in FEP incidence than the prevalence of daily or high-potency cannabis use between settings.

**Study Design:**

We used incidence data in people aged 18–64 years from 14 settings of the EU-GEI study. We estimated the prevalence of daily and high-potency cannabis use in controls as a proxy for usage in the population at-risk; multiple imputations by chained equations and poststratification weighting handled missing data and control representativeness, respectively. We modeled FEP incidence in random intercepts negative binomial regression models to investigate associations with the prevalence of cannabis use in controls, unemployment, and owner-occupancy in each setting, controlling for population density, age, sex, and migrant/ethnic group.

**Study Results:**

Lower owner-occupancy was independently associated with increased FEP (adjusted incidence rate ratio [aIRR]: 0.76, 95% CI: 0.61–0.95) and non-affective psychosis incidence (aIRR: 0.68, 95% CI: 0.55–0.83), after multivariable adjustment. Prevalence of daily cannabis use in controls was associated with the incidence of affective psychoses (aIRR: 1.53, 95% CI: 1.02–2.31). We found no association between FEP incidence and unemployment or high-potency cannabis use prevalence. Sensitivity analyses supported these findings.

**Conclusions:**

Lower setting-level owner-occupancy and increased prevalence of daily cannabis use in controls independently contributed to setting-level variance in the incidence of different psychotic disorders. Public health interventions that reduce exposure to these harmful environmental factors could lower the population-level burden of psychotic disorders.

## Introduction

The incidence of first-episode psychosis (FEP) varies geographically.^1–6^ For example, an earlier publication from the “EUropean Network of National Schizophrenia Networks Studying Gene-Environment Interactions” (EU-GEI) study from our group detected an 8-fold variation in the incidence of FEP across 17 settings in 6 countries.^2^ In that article, we found that this variation was strongly associated with the proportion of the population in each setting who owned their own home, with greater levels of homeownership associated with lower FEP rates.^2^ This may be a marker of socioeconomic position since settings with lower levels of homeownership are—on average—likely to be more socioeconomically deprived than others. Most studies have found that greater neighborhood social deprivation is associated with a higher incidence of psychotic disorders,^7^ although some have observed that this disappeared after controlling for other individual and setting-level variables,^7^ such as population density and having foreign-born parents.^8^ Higher unemployment has also been linked to a higher incidence of schizophrenia^9^ and non-affective psychotic disorders, but not of affective psychotic disorders.^10^ Finally, settings with higher levels of homeownership also tend to be less socially transient, as homeowners move house less frequently,^11^ and areas that are more socially fragmented have been observed to experience higher psychosis rates.^12^

In a later EU-GEI study, Di Forti et al^13^ reported a strong ecological correlation between the proportion of controls who reported daily or high-potency cannabis use and the age-sex-ethnicity standardized incidence of psychotic disorders between settings. This suggests that the prevalence of daily and high-potency cannabis use among controls, as proxies for the prevalence of cannabis use at the population level (hereafter: setting-level cannabis use), is a determinant of the incidence of psychotic disorders, but this issue has received less attention to date than the consistent individual-level associations observed between cannabis use and psychosis risk.^14,15^ Over the past decades, cannabis use^16^ (and cannabis use disorder^17^) has increased in European populations, and a UK-based study reported a 15% annual increase in the incidence of substance-induced psychoses between 1978 and 1999^18^; model projections^16^ have also estimated that increases in the incidence of all psychotic disorders of up to 29% would have been apparent by 2010, assuming causality. Empirical data on this issue have only recently begun to emerge. For example, nationwide data from Denmark suggest that an increase in cannabis use disorder has co-occurred alongside an increase in schizophrenia cases and that cannabis use disorder may—assuming causality—account for 15% of recent cases of schizophrenia in males, and 4% in females.^17,19^

Despite this initial evidence, neither of the aforementioned EU-GEI studies^2,13^ simultaneously controlled for the potential confounding effects of setting-level cannabis use and social deprivation, or other potentially relevant area-level confounders such as population density.^1^ This is important because cannabis use could be at least partially socially determined, meaning its association with psychotic disorders incidence could be confounded by common causes such as deprivation. For example, recent findings from 3 settings in the Global South, as part of the INTREPID study, also reported that incidence rates were highest in the setting with the highest prevalence of frequent cannabis use in controls^20^; this setting, however, also had high levels of other forms of social adversity including crime.^21^ Clarifying whether levels of cannabis use in the general population are associated with increased incidence of psychotic disorders, and the extent to which this may be confounded by other socioenvironmental factors, is essential for both etiological research and informing public mental health. We therefore reanalyzed incidence data from the EU-GEI study to investigate the relative contribution of several setting-level socioenvironmental risk factors, including cannabis use, in explaining variation in FEP incidence. We hypothesized that after controlling for relevant confounders, setting-level deprivation (operationalized as owner-occupancy and unemployment) would be more strongly associated with variation in the incidence of psychotic disorders than setting-level daily and high-potency cannabis use, given the potential for confounding introduced above, although we reasoned both may have independent effects.

## Methods

### Participants and Study Design

The EU-GEI incidence and case-control study included FEP patients and population-based controls from 6 countries (Brazil, France, Italy, Spain, the Netherlands, and the United Kingdom) across 17 settings (supplement 1).^22^ Between May 2010 and April 2015, all people aged 18–64 years diagnosed for the first time with a nonorganic untreated FEP (International Classification of Diseases [ICD]-10: F20–33) were included in the incidence sample if resident in the catchment area at first presentation. Those diagnosed with psychosis due to an organic condition (F09) or psychoactive substance use (F1x.5), who had IQ <50 or intellectual disability (F70–79), or who had previously been in contact with mental health services for psychotic symptoms were excluded. Incident cases were also invited to participate in the case-control study. In the present study, we used control data to estimate the setting-level proportion of cannabis use in the population (see below). Controls were recruited from the general population via a combination of random and quota sampling.^22^ We used the most accurate demographic data available from each catchment area to set quotas for controls, aiming to select samples that broadly represented the age, sex, and ethnic groups of the local population at-risk. Next, these quotas were filled by using (stratified) random sampling from lists of all postal addresses and via general practitioner lists from randomly selected surgeries, and by ad hoc approaches such as adverts and leaflets. Potential controls were eligible if they had no history of psychotic disorder, had sufficient knowledge of the local language, resided in the catchment area, and were aged 18–64 years. Controls provided written informed consent. Ethical approval was obtained from local research ethics committees. All study procedures followed local and (inter)national ethical standards, including the Declaration of Helsinki.

We excluded the Puy-de-Dôme setting due to missing data on migrant and ethnic groups, the Paris setting due to no control recruitment, and the Veneto setting due to cannabis data quality issues, resulting in a dataset with 14 settings (supplement 1).

### Population At-risk

We estimated the population at-risk in each setting using demographic data from national or regional statistics institutions (see supplement 1), stratified by age (5-year bands, except 18–24 years), sex (male, female), and migrant/ethnic group. The latter was dichotomized into a binary indicator of ethnic minority or migrant group vs ethnic majority or nonmigrant group (henceforth, migrant/ethnic minority or majority groups) in each country, following the official classification used in each jurisdiction (see supplement 1 and Jongsma et al^2^ for further details). The population at-risk was multiplied by the case ascertainment duration in years in each setting to estimate person-years at-risk.

### Measures

#### Outcomes

Our primary outcome was the incidence of nonorganic psychotic disorders (ICD-10: F20–33). We also included the incidence of non-affective psychotic disorders (F20–29) and affective psychotic disorders (F30–33) as secondary outcomes. Full details of the diagnostic procedure are provided in supplement 1.

#### Individual-Level Covariates

We used the Medical Research Council Sociodemographic Questionnaire^23^ and case notes to collect information on age-at-first-contact, sex, and migrant/ethnic group (coded as before).

#### Setting-Level Exposures and Covariates

We estimated the proportion of daily and high-potency cannabis users among controls in each setting as proxies for the prevalence of setting-level cannabis use in the population at-risk using the modified Cannabis Experiences Questionnaire (CEQ) (supplement 1).^13,24,25^ This is based on several assumptions that we explore in this article, including that our controls are representative of the population at-risk from which cases arise in terms of their patterns of cannabis consumption. The prevalence of daily cannabis use in controls in each setting was calculated by dividing the number of controls who reported daily cannabis use by the total number of controls who completed this CEQ item. Prevalence of high-potency cannabis use in controls in each setting was calculated similarly and defined as self-reported lifetime use of cannabis types with greater than or equal to 10% concentration of Δ^9^-tetrahydrocannabinol (THC), cannabis’ dominant psychoactive molecule,^26^ following our previous approach.^13^ Given the potential for missing cannabis use data in the controls to influence these prevalence estimates (supplement 2), we first imputed missing values for daily and high-potency cannabis use in controls using multiple imputations by chained equations, including a large number of auxiliary variables potentially relevant to the imputation of missing cannabis use values (supplement 2). We ran 30 imputations and derived estimates of the imputed proportion of daily and high-potency cannabis use in each setting, combined across these imputed datasets according to Rubin’s Rule. Finally, we applied poststratification weighting to these estimates so that they were representative of the age (18–24, 25–34, 35–64 years), sex (men, women), and broad migrant/ethnic structure (majority, minority) of the underlying population at-risk in each setting from which controls were drawn.

The proportion of the economically active population who were unemployed and the proportion of owner-occupied homes in each setting were obtained from the 2011 European Household and Population Census and the 2010 Brazil Census.^27,28^ Population density, defined as people per square kilometer, was derived from population estimates from national statistics institutions. All setting-level variables were *Z*-standardized to have a mean of 0 and SD of 1, to enable estimation of comparable effect sizes during modeling. We estimated Spearman correlation coefficients between setting-level variables.

### Statistical Analysis

We reported basic descriptive statistics on all individual-level (age, sex, and migrant/ethnic group) and setting-level variables (prevalence of daily and high-potency cannabis use in controls in each setting, unemployment, owner-occupancy, and population density) by setting, and compared incidence cases to person-years at-risk using Pearson’s chi-square (χ^2^) goodness of fit tests. Similarly, we compared the representativeness of controls to the population at-risk per setting by age, sex, and migrant/ethnic group.

We modeled incidence rates using random intercepts negative binomial regression (supplement 4). First, we quantified setting-level variation in FEP incidence in null (without covariates, ie, a variance-component model) and fully adjusted models. Second, we estimated incidence rate ratios (IRRs) and 95% CIs in univariable models to investigate crude associations between FEP incidence and each setting-level variable. Third, we fitted multivariable models to mutually adjust for all setting-level variables and individual-level age, sex, their interaction, and migrant/ethnic group. This process was repeated for our secondary outcomes. Finally, we conducted 3 sensitivity analyses to understand whether our modeling choices affected the results. First, we compared our main incidence findings to those derived based on complete cannabis use data in controls. Second, we compared our main findings to those from analyses restricted to a subset of 10 of the 11 settings included in Di Forti et al,^13^ which first reported strong correlations between cannabis use prevalence in controls and FEP incidence. In this second sensitivity analysis, we reran our models based on our multiply imputed, weighted cannabis use estimates, while adjusting for all setting-level covariates. In our third sensitivity analysis, we replicated the correlational results presented in Di Forti et al^13^ in a model-based framework. Consistent with that article, we restricted multivariable adjustment to age, sex, and ethnicity in these 10 settings, using prevalence estimates of daily and high-potency cannabis use in controls based on complete cannabis data without poststratification weighting. We could not include data from 1 of the 11 settings (Puy-de-Dôme) in these final 2 sensitivities due to entirely missing incidence data by migrant/ethnic group in that location. All analyses were conducted in Stata MP/17.0.^29^

## Results

### Sample Characteristics

The original incidence sample^2^ included 2774 individuals, of whom we excluded 271 (9.8%) from Paris, Puy-de-Dôme, and Veneto. From the remaining 2503 cases, we excluded 43 (1.7%) participants due to missing or inconsistent data on age, sex, and/or migrant/ethnic group. Our final incidence sample comprised 2460 individuals diagnosed with FEP (1080 women; 43.9%), of whom 1819 were diagnosed with non-affective psychotic disorder (73.9%; 732/1819 women [40.2%]), 611 with affective psychotic disorder (24.8%; 335/611 women [54.8%]), and 30 with psychotic disorder not otherwise specified (1.2%; 13/30 women [43.3%]). Most cases were younger than 35 years old (1519; 61.7%) and from the nonmigrant/ethnic majority group (1461; 59.4%; table 1 and supplement 5). There was a higher proportion of male cases in the younger age groups and a higher proportion of female cases in the older age groups (supplement 6).

**Table 1.** Characteristics of the Incidence Sample and the Setting-Level Variables per Catchment Area

Catchment Area	Incidence Sample				Setting-Level Variables				
	All FEP				In Controls		In Population		
	Cases, *N* (% of Total Across Catchment Areas)	Women, *N* (%)^a^	Age-at-First-Contact <35 (y), *N* (%)^a^	Migrant/Ethnic Minority Groups,*N* (%)^a^	Daily Cannabis, %^b^	High-Potency Cannabis, %^b^	Unemployment, %	Owner-Occupancy, %	Population Density, People per km^2^
England									
Southeast London	262 (10.7)	121 (46.2)	144 (55.0)	201 (76.7)	12.5	26.5	5.3	35.0	6162.3
Cambridgeshire	266 (10.8)	115 (43.2)	187 (70.3)	103 (38.7)	3.4	9.5	3.0	67.0	241.5
The Netherlands									
Amsterdam	293 (11.9)	104 (35.5)	175 (59.7)	204 (69.6)	14.8	54.3	4.0	46.3	4908.0
Gouda and Voorhout^c^	166 (6.7)	65 (39.2)	111 (66.9)	40 (24.1)	4.6	19.2	4.3	58.7	4208.0
Spain									
Madrid	88 (3.6)	30 (34.1)	51 (58.0)	12 (13.6)	14.4	13.7	13.0	76.8	4997.2
Barcelona	108 (4.4)	46 (42.6)	78 (72.2)	26 (24.1)	15.5	19.9	14.0	74.3	12 326.5
Valencia^c^	59 (2.4)	27 (45.8)	38 (64.4)	10 (16.9)	10.3	12.0	17.7	82.7	14 468.0
Oviedo^c^	82 (3.3)	42 (51.2)	44 (53.7)	15 (18.3)	3.2	4.3	13.1	79.9	141.9
Santiago^c^	36 (1.5)	15 (41.7)	18 (50.0)	1 (2.8)	0.0	0.0	13.8	77.9	102.2
Cuenca^c^	27 (1.1)	6 (22.2)	20 (74.1)	7 (25.9)	2.7	3.8	17.0	81.9	11.6
France									
Val-de-Marne	210 (8.5)	102 (48.6)	124 (59.0)	67 (31.9)	11.9	20.4	4.2	47.6	3721.3
Italy									
Bologna^c^	165 (6.7)	79 (47.9)	107 (64.8)	49 (29.7)	1.1	8.5	3.2	71.4	2744.0
Palermo	179 (7.3)	79 (44.1)	114 (63.7)	22 (12.3)	3.4	4.4	8.3	70.2	4200.0
Brazil									
Ribeirão Preto	519 (21.1)	249 (48.0)	308 (59.3)	242 (46.6)	7.2	2.1	4.4	80.8	145.2
Total	2460 (100)	1080 (43.9)	1519 (61.7)	999 (40.6)	NA	NA	NA	NA	NA
Mean^d^ (SD)	NA	NA	NA	NA	7.2 (5.7–8.7)	12.2 (10.4–14.0)	9.0 (5.5)	67.9 (15.2)	4169.8 (4486.8)

*Note*: FEP, first-episode psychosis; IQR, interquartile range; NA, not applicable.

^a^Per catchment area.

^b^Following multiple imputation by chained equations (supplement 2) to impute missing cannabis use data among controls, with poststratification weights applied (supplement 3).

^c^Excluded from 8-setting analyses, which were restricted to settings with a maximum of 10% missing data on cannabis use in the control sample.

^d^Setting-level mean and SD, except cannabis use variables which show weighted mean cannabis use in controls and 95% CIs following multiple imputation across imputed datasets.

Cases were identified during 11.9 million person-years (broken down by setting, age, sex, and migrant/ethnic group in supplement 7), equivalent to a crude incidence rate of 20.6 per 100 000 person-years (95% CI: 19.8–21.4). The crude incidence of non-affective psychotic disorders was 15.2 per 100 000 person-years (95% CI: 14.6–16.0) and the crude incidence of affective psychotic disorders was 5.1 per 100 000 person-years (95% CI: 4.7–5.5) (supplement 6). Compared with person-years at-risk, a higher proportion of cases were men (χ^2^(1) degree of freedom [df]: 43.5, *P* < .001), younger (χ^2^(8) df: 825.16, *P* < .001) and from migrant/ethnic minority groups (χ^2^(1) df: 428.1, *P* < .001; supplement 8).

### Setting-Level Variable Characteristics

Of 1335 controls, 1304 (97.7%) had complete data on daily and/or high-potency cannabis use. Proportions of missing cannabis use data per setting ranged from 0% to 36% (supplement 2). Our controls were, on average, younger and less likely to be from the nonmigrant/ethnic majority group compared with the population at-risk (*P* < .001; supplement 3). Following multiple imputation poststratification weighting, the proportion of controls who reported daily cannabis use ranged from 0.0% (Santiago, Spain) to 15.5% (Barcelona, Spain), with a weighted mean of 7.2% (95% CI: 5.7–8.7). High-potency cannabis use ranged from 0.0% (Santiago, Spain) to 54.5% (Amsterdam, the Netherlands), with a weighted mean of 12.2% (95% CI: 10.4–14.0). Of 1320 controls with non-missing information on current cannabis use (98.9%), 150 (11.4%) reported current use (supplementary table 2ii).

Unemployment rates ranged from 3.0% of the population (Cambridgeshire, United Kingdom) to 17.7% (Valencia, Spain). Owner-occupancy rates ranged from 35.0% (Southeast London, United Kingdom) to 82.7% (Valencia, Spain). Population density ranged from 11.6 (Cuenca, Spain) to 14 468.0 (Valencia, Spain) people per km^2^. Additional descriptive statistics of the setting-level variables are provided in supplement 9.

We observed statistically significant (*P* < .05) setting-level correlation between the prevalence of high-potency cannabis use and daily cannabis use (*r*_s_ = .79, *P* = .001), prevalence of high-potency cannabis use and owner-occupancy (*r*_s_ = −.67, *P* = .008), unemployment and owner-occupancy (*r*_s_ = .70, *P* = .006), prevalence of daily cannabis use and population density (*r*_s_ = .78, *P* = .001), and prevalence of high-potency cannabis use and population density (*r*_s_ = .71, *P* = .005) in our 14-setting dataset.

### Multilevel Modeling of Psychotic Disorder Incidence

**Fig. 1.**
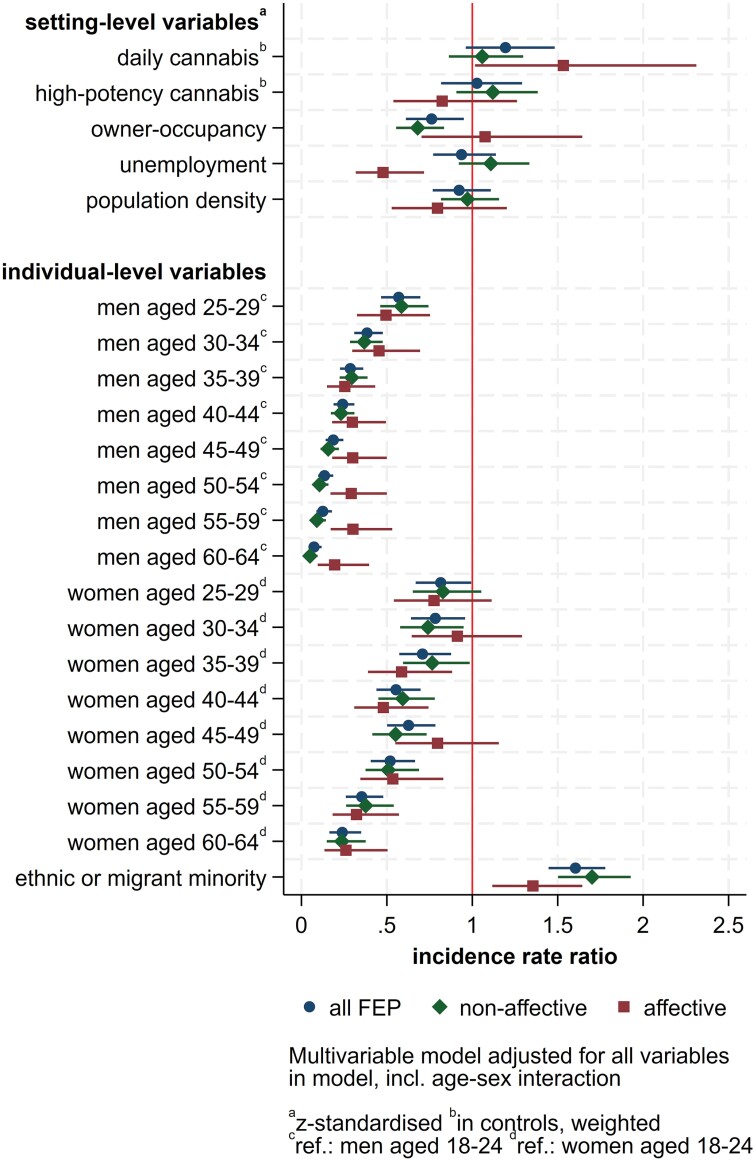
Multivariable random intercepts negative binomial regression of the association between sociodemographic and socioenvironmental variables and all FEP, non-affective psychotic disorders, and affective psychotic disorders incidence. *Note*: FEP, first-episode psychosis.

Null models provided evidence of substantial variation in incidence rates for all outcomes, which was attenuated but remained in fully adjusted models (supplement 11). Following multivariable modeling (table 2, figure 1, supplement 11), we observed an independent effect of setting-level owner-occupancy on FEP incidence, with a 1 SD increase in owner-occupancy associated with a 25% decrease in FEP incidence (IRR: 0.76, 95% CI: 0.61–0.95, equivalent to an IRR of 1.32, 95% CI: 1.05–1.64 for a 1 SD decrease in owner-occupancy). We found univariable evidence that the prevalence of daily (IRR: 1.34, 95% CI: 1.04–1.73) and high-potency cannabis use in controls in each setting (IRR: 1.50, 95% CI: 1.23–1.82) were associated with increased FEP incidence. This was attenuated to the null with respect to the prevalence of daily (IRR: 1.19, 95% CI: 0.96–1.48) and high-potency cannabis (IRR: 1.03, 95% CI: 0.82–1.29) use in controls after multivariable modeling. No other setting-level variables were associated with FEP incidence in our fully adjusted model.

**Table 2.** Univariable and Multivariable Random Intercepts Negative Binomial Regression of the Association Between Setting-Level Characteristics and All FEP, Non-affective Psychotic Disorders, and Affective Psychotic Disorders Incidence

Setting-Level Variable^a^	All FEP		Non-affective Psychotic Disorders		Affective Psychotic Disorders	
	Univariable IRR (95% CI)	Multivariable IRR (95% CI)^c^	Univariable IRR (95% CI)	Multivariable IRR (95% CI)^c^	Univariable IRR (95% CI)	Multivariable IRR (95% CI)^c^
% owner-occupancy	**0.65 (0.56–0.75)**	**0.76 (0.61–0.95)**	**0.63 (0.55–0.72)**	**0.68 (0.55–0.83)**	**0.65 (0.43–0.99)**	1.07 (0.70–1.64)
% daily cannabis (controls)^b^	**1.34 (1.04–1.73)**	1.19 (0.96–1.48)	**1.38 (1.07–1.78)**	1.06 (0.86–1.30)	1.18 (0.72–1.92)	**1.53 (1.02–2.31)**
% high-potency cannabis (controls)^b^	**1.50 (1.23–1.82)**	1.03 (0.82–1.29)	**1.56 (1.31–1.87)**	1.12 (0.91–1.38)	1.39 (0.88–2.20)	0.82 (0.54–1.26)
% unemployment	**0.73 (0.58–0.92)**	0.94 (0.77–1.14)	0.79 (0.60–1.03)	1.11 (0.92–1.33)	**0.48 (0.35–0.65)**	**0.48 (0.32–0.72)**
Population density (people per km^2^)	1.04 (0.78–1.38)	0.92 (0.77–1.11)	1.12 (0.84–1.49)	0.97 (0.82–1.16)	0.78 (0.49–1.24)	0.80 (0.53–1.20)

*Note*: FEP, first-episode psychosis; IRR, incidence rate ratio.

Bold: *P* < .05 statistically significant.

^a^ *Z*-standardized; IRR associated with 1 SD changes.

^b^Setting-level proportion of daily- or high-potency cannabis use reported by controls with multiple imputation and poststratification weights applied (see supplements 2 and 3 for methods).

^c^Adjusted for age, sex, age-sex interaction, migrant/ethnic group, and all other variables in the table.

For non-affective psychotic disorders, only lower owner-occupancy (IRR: 0.68, 95% CI: 0.55–0.83) was associated with greater incidence following multivariable modeling. Univariable associations between non-affective psychotic disorders incidence and the prevalence of daily (IRR: 1.38, 95% CI: 1.07–1.78) and high-potency (IRR: 1.56, 95% CI: 1.31–1.87) cannabis use in controls did not persist in fully adjusted models (IRR_daily_: 1.06, 95% CI: 0.86–1.30; IRR_highpot_: 1.12, 95% CI: 0.91–1.38). For affective psychotic disorders, we found an association between greater prevalence of daily cannabis use in controls and higher incidence rates (IRR: 1.53, 95% CI: 1.02–2.31), but no evidence of variation in incidence by setting-level owner-occupancy (IRR: 1.07, 95% CI: 0.70–1.64) or prevalence of high-potency cannabis use in controls (IRR: 0.82, 95% CI: 0.54–1.26; table 2). Unexpectedly, greater unemployment was associated with a decreased incidence of affective psychotic disorders in multivariable models (IRR: 0.48, 95% CI: 0.32–0.72).

### Sensitivity Analyses

In our first sensitivity analysis, our main results were comparable to those found when we substituted the imputed cannabis use prevalence variables for those derived using complete cannabis data only in the controls (supplement 12, “CC” results in supplementary table 12i). In our second sensitivity analysis, our main results with respect to owner-occupancy, unemployment, and population density were comparable to those in analyses restricted to 10 settings included in an earlier publication by Di Forti et al^13^ with less than 10% missing data on daily cannabis use (see Methods and supplement 12). We note 2 differences in this sensitivity analysis vs our main results. First, a positive association between *greater* prevalence of daily cannabis use and *higher* incidence rates of all FEP (IRR: 1.31, 95% CI: 1.10–1.55, “MI_10_” results in supplementary table 12i); this new finding was apparently driven by an association between the prevalence of daily cannabis use and incidence of affective psychotic disorders (IRR: 1.97, 95% CI: 1.33–1.92), but not non-affective psychotic disorders (IRR: 1.04, 95% CI: 0.84–1.29) in these 10 settings (consistent with our main findings). Second, we noted a *negative* association between a *greater* prevalence of high-potency cannabis use in controls and a *lower* incidence of affective psychotic disorders (MI_10_: IRR: 0.70, 95% CI: 0.49–0.99). Our final sensitivity analysis closely replicated Di Forti et al’s^13^ correlational findings by rerunning models in these 10 settings limited to adjustment for age, sex, and ethnicity only, and using unweighted, cannabis prevalence estimates based on complete data. In contrast to our main analyses, and consistent with Di Forti et al,^13^ we observed strong associations between the prevalence of daily (IRR: 1.46, 95% CI: 1.17–1.82) and high-potency (IRR: 1.37, 95% CI: 1.14–1.66) cannabis use and incidence of all FEP, after adjustment for age, sex, and ethnic group only (“CC10” results in supplementary table 12i). Similar results were found with respect to non-affective psychotic disorders (supplementary table 12i).

## Discussion

We observed that lower setting-level owner-occupancy was independently associated with increased FEP incidence, including non-affective psychotic disorders, across settings in Europe and Brazil, which persisted after adjustment for setting-level daily and high-potency cannabis use, unemployment, population density, and individual-level age, sex, and migrant/ethnic group. We found evidence of an association between a greater prevalence of daily cannabis use in controls and the incidence of affective psychotic disorders, after multivariable modeling, but no such evidence with respect to all FEP or non-affective psychotic disorders, and no evidence of any association between psychotic disorders incidence and the prevalence of high-potency cannabis use in controls.

### Meaning of the Findings

The association between cannabis use and increased psychosis risk for individuals is well established,^14^ and may be partly causal,^13,30^ with evidence that THC has psychotogenic effects on the human brain.^31,32^ It is unclear, however, whether the prevalence of cannabis use within a population leads to increased rates of psychotic disorders at the population level, a possibility raised by strong correlations between the proportion of daily and high-potency cannabis use in controls and age-, sex-, and ethnicity-adjusted incidence rates in 11 EU-GEI settings reported in a previous publication from our group.^13^ Here, we formally investigated those correlations, using multilevel modeling to consider the role of other setting-level confounders, including markers of deprivation, and recovering missing cannabis use data in controls via multiple imputation to extend the study into 14 settings, where we weighted prevalence estimates of cannabis use in controls to reflect the basic sociodemographic profile of the underlying population at-risk. Under this approach, cannabis use appeared to have a smaller effect on population-level incidence than markers of deprivation in our models of all FEP and non-affective psychotic disorders.

In line with previous literature,^2,33,34^ we found strong evidence that the incidence of all FEP and non-affective psychotic disorders was lower in areas where more people owned their house. Secure, stable, and affordable housing is fundamental to health,^35^ and as a potential marker of social deprivation, there is evidence that this is a predictor of psychosis incidence.^7^ Owner-occupancy may also be a proxy for wider socioenvironmental exposures including social fragmentation, which has been associated with increased psychosis rates,^12^ and social status.^36,37^ More socially fragmented areas may be more tolerant of perceived deviant behaviors in their communities, including antisocial acts, crime, or substance abuse. As such, we posited that levels of owner-occupancy and unemployment across our settings may have been common causes of the association between the prevalence of cannabis use and the incidence of psychotic disorders at the population level.

We found no evidence that the prevalence of daily or high-potency cannabis use in controls was associated with setting-level incidence rates of all FEP and non-affective psychotic disorders after adjustment for other important setting-level variables and accounting for missing cannabis use data and sample representativeness of controls. We did, however, observe an association between the prevalence of daily (but not high-potency) cannabis use in controls in each setting and the incidence of affective psychotic disorders. Further research is required to replicate these findings, and better understand whether and why cannabis use may contribute to population-level rates of some, but not all psychotic disorders. One possibility is that our results imply that other setting-level factors—most notably, owner-occupancy as a marker of socioeconomic deprivation and/or social fragmentation—may be common causes of univariable associations between the prevalence of daily/high-potency cannabis use and the incidence of psychotic disorders. That is, social disadvantage causes both higher levels of cannabis use and higher incidence rates. It is also possible that population-level cannabis use may mediate the relationship between social disadvantage and psychotic disorder incidence, but longitudinal data would be required to validate this.

These findings are consistent with Geoffrey Rose’s subtle, but critical appreciation of how individual risk translates to overall population health^38^; although individuals who smoke high-potency cannabis are at great risk of psychotic disorder,^13,25^ one might be able to prevent more cases in certain settings by concentrating preventive efforts on reducing daily use and/or ameliorating deprivation in the population. The exact strategy required may vary by setting since the prevalence and impact of daily and high-potency cannabis use is greater in some populations (eg, Amsterdam, Southeast London) than in others.^13^ Indeed, we observed stronger associations between the prevalence of daily cannabis use in controls and the incidence of all FEP in a sensitivity analysis restricted to 10 of 11 settings included in Di Forti et al^13^ with no more than 10% missing cannabis use data. All 4 excluded settings from these analyses were in Spain (supplementary table 12ii) and had lower than average daily cannabis use, suggesting that cannabis use may only have detectable effects on the incidence of psychotic disorders above certain prevalence thresholds in the population. Similar threshold effects have been observed for deprivation,^39,40^ and this may provide an important line of inquiry for guiding preventive psychiatry and public mental health. While efforts to reduce deprivation would improve public health across multiple domains, not limited to psychosis, achieving multisectoral, interdisciplinary, and meaningful traction on this issue is complex and obdurate. Therefore, in the shorter term, public mental health should concentrate on any preventive efforts to reduce cannabis use in selective or indicated groups where the individual risk of psychosis due to cannabis use is highest. This may include those living in deprived areas, high-potency users or people with preexisting mental health vulnerabilities, and may offer more amenable, immediate directions for intervention strategies in public mental health.

Our findings concerning (greater) unemployment and population density were inconsistent and sometimes revealed unexpected^1,9,40–42^ associations with (lower) incidence of psychotic disorders. This suggests that, particularly for population density, its long-established association with greater psychosis incidence in mainly Northern European studies^1,42^ does not apply in all contexts, as has been suggested,^21,43^ and may be driven by other socioenvironmental factors such as income, inequality, or social capital.

### Strengths and Limitations

Our study included a large sample from multiple countries and settings that varied in their social contexts and sociodemographic characterization. We were also able to control for several important theoretically driven confounders. We applied poststratification weights to the setting-level cannabis variables to make them representative of our at-risk populations by age, sex, and broad migrant/ethnic group. We also recovered missing cannabis use data in controls to allow accurate estimation of the prevalence of daily and high-potency cannabis use across 14 settings. Our results based on multiply imputed data (using a comprehensive range of auxiliary variables) were similar to those using complete data only in controls across these 14 settings in a sensitivity analysis, suggesting that missing data patterns did not substantially bias our findings. We conducted additional sensitivity analyses restricted to a subset of 10 of the 11 settings in the Di Forti et al^13^ article that first reported strong correlations between the unweighted prevalence of daily and high-potency cannabis use in controls (based on complete data only) and psychosis incidence. We were able to replicate those correlations under a model-based approach in a naive analysis (CC_10_; supplementary table 12i), before multiple imputation, poststratification weighting and adjustment for other important setting-level covariates. Use of these techniques in our main analyses provided greater insight into the relative contribution of several setting-level covariates on incidence of psychotic disorders across the broadest range of settings possible in this study. We also used reliable denominator data and well-validated measures, and the controls were population based.

Nevertheless, our results should be interpreted alongside several limitations. First, setting-level cannabis information was recorded from control participants in the EU-GEI study. Although we used poststratification weights to account for basic sociodemographic differences with the population at-risk, we do not know the extent to which this made controls representative of cannabis behaviors in the population at-risk in each setting, because information on cannabis use was not available in our denominator population. We attempted to minimize potential selection bias by not mentioning cannabis in documents used to recruit controls. Second, the relatively small number of controls in some settings (supplement 4) may have increased uncertainty around estimates of cannabis use in the population at-risk. Third, although we used a validated questionnaire to measure cannabis use, recall bias cannot be excluded. Fourth, although we controlled for broad migrant/ethnic group, this was restricted to a binary definition given the heterogeneity in definitions and population structures between countries; this may have led to some residual confounding. Fifth, we were unable to control for all potentially relevant individual-level risk factors such as genetic liability^44^ or childhood adversities,^45^ because these data were not available for our denominator population while estimating incidence. Sixth, we found little evidence of an association between psychosis incidence and the prevalence of high-potency cannabis use. We used a 10% THC cutoff to define high-potency cannabis use, but this may have been too conservative in some settings (although a 15% cutoff would have provided the same results). For example, in the Netherlands between 2010 and 2015, herbal cannabis contained up to 17.8% THC, and cannabis resin even 35.0%.^46^ We may therefore have differentially misclassified levels of high-potency cannabis use in some settings. Finally, our high-potency cannabis variable measured lifetime use, and did not indicate frequency or ongoing use.

## Conclusions

We found that lower owner-occupancy was independently associated with a higher incidence of all FEP and non-affective psychotic disorders across all analyses, while the prevalence of daily cannabis use was independently associated with the incidence of affective psychotic disorders. This extends our previous understanding of the epidemiology of psychotic disorders by illuminating how the setting-level expression of FEP is shaped by various socioenvironmental exposures, and suggests that policymakers focus on targeting public mental health interventions that seek to ameliorate exposure to deprivation, housing instability, and cannabis use via universal and selective primary prevention strategies that improve the population distribution of exposure to adverse social environments.

## Supplementary Material

Supplementary material is available at https://academic.oup.com/schizophreniabulletin/.

sbae072_suppl_Supplementary_Material

## Data Availability

A preprint of this manuscript and associated analytical scripts and log files can be accessed at https://osf.io/98uqk/.
