# Supplementary material for: The Role of Social Deprivation and Cannabis Use in Explaining Variation in the Incidence of Psychotic Disorders: Findings From the EU-GEI Study
Source: Schizophr Bull. 2024 May 24;50(5):1039–49. doi: 10.1093/schbul/sbae072 (PMC11349009; doi:10.1093/schbul/sbae072)
Supplement: sbae072_suppl_Supplementary_Material [file sbae072_suppl_supplementary_material.docx]

**Supplementary materials**

1. Additional information on case ascertainment, denominator source and type, control recruitment, and variables
2. Further information on cannabis use in controls per setting, missing data, and multiple imputation by chained equations
3. Control representativeness to the population at-risk per setting and post-stratification weighting
4. Additional information on the statistical analyses
5. Sample characteristics for non-affective and affective psychotic disorders
6. Overall sample characteristics of incidence cases
7. Overall sample characteristics for the person-years at-risk
8. Comparison of the incidence cases with the person-years at-risk
9. Descriptive statistics of setting-level variables
10. Correlation matrices and scatterplots of setting-level variables
11. Full model parameter estimates from negative binomial multilevel modelling
12. Sensitivity analyses
13. Collaborators
14. References

**Supplement 1: Additional information on case ascertainment, denominator source and type, control recruitment, and variables**

*Case ascertainment*

In the Veneto region, case ascertainment took place between January 2005 and December 2007, and an upper age limit of 54 instead of 64 years was applied.

*Supplemental Table 1i: Denominator source and type by country*

|  | **Denominator source (year)** | **Denominator type** |
| --- | --- | --- |
| **Brazil** | Sistema IBGE de Recuperação Automática (2010) | yearly estimates |
| **France** | Institute Nationale de la Statisique et des Études Économiques (2011) | yearly estimates |
| **Italy** | L’Instituto Nazionale di Statistica (yearly) | yearly estimates |
| **Spain** | Instituto Nacional de Estadística (2012) | yearly estimates |
| **The Netherlands** | Statistics Netherlands (2014) | yearly estimates |
| **The United Kingdom** | Office for National Statistics (2011) | census |

*Supplemental Table 1ii: Migrant/ethnic minority and majority groups classification by country*

|  | **Non-migrant or ethnic majority group** | **Migrant or ethnic minority group** |
| --- | --- | --- |
| **Brazil** | Ethnic group is White | Ethnic group is Black, Asian, Mixed, Indigenous, or not declared |
| **France** | Country of birth is Metropolitan France and Overseas France (in French: *les départements et regions d’outre-mer*) | Country of birth is countries in the European Union (excluding France), other European countries and Turkey, the Maghreb, other African countries, or other countries |
| **Italy** | Country of birth is Italy | Country of birth other than Italy |
| **Spain** | Country of birth is Spain | Country of birth other than Spain |
| **The Netherlands** | Individual and both parents were born in the Netherlands | Individual and/or at least 1 parent was born outside of the Netherlands |
| **The United Kingdom** | Ethnic group is White British | Ethnic group is White Irish, White Traveler or Gypsy, White Other, Mixed White & Black Caribbean, Mixed White & Black African, Mixed White & Asian, Mixed other, Indian, Pakistani, Bangladeshi, Chinese, Other Asian, Black African, Black Caribbean, Black Other, Arabic, or other |

*Daily and high-potency cannabis use*

Frequency of cannabis use was determined from the Cannabis Experiences Questionnaire (CEQ) item: “Describe how often [you have used cannabis in your lifetime] from the following options: a) I used it only once or twice; b) about once a year; c) few times a year; d) about once/twice a month; e) about once a week; f) more than once a week; g) every day”. High-potency cannabis use was estimated from the item: “What type of cannabis did you mostly use?”, which participants answered in their native language using colloquial names (Di Forti 2019). Δ⁹-tetrahydrocannabinol (THC) values corresponding to these cannabis types were retrieved from the European Monitoring Centre for Drugs and Drug Addiction 2016 report, and national reports and publications (European Monitoring Centre for Drugs and Drug Addiction, Spanish Ministry of Health and Consumer Affairs 2012, European Monitoring Centre for Drugs and Drug Addiction 2016, Niesink 2013, Niesink 2015, Zamengo 2015, Observatoire Français des Drogues et des Toxicomanies (ODFT) 2015, Lopes de Oliveira 2008, Potter 2008, Hardwick 2008). We did not corroborate the CEQ data with blood, urine, saliva, or hair samples, because this would only provide information about recent use, whilst our cannabis variables examined lifetime use.

*Unemployment and owner-occupancy*

The proportion of the economically active population who were unemployed and the proportion of owner-occupied homes were measured at the Nomenclature of Territorial Units for Statistics (NUTS)-2 level for the European settings. For the Brazil setting, this was measured at the Federative Units level.

*Outcomes*

Where possible, diagnoses were derived based on standardized operational criteria using the Operational Criteria Checklist algorithm (OPCRIT) (Rucker 2011). If an OPCRIT diagnosis could not be obtained, the clinical diagnosis was used instead (for details see Jongsma 2018). Where no precise diagnosis could be obtained, we retained cases in the FEP analysis model but excluded them from the non-affective and affective psychotic disorders models. Inter-rater reliability of the OPCRIT measurement was good (Kappa=0.7) (Gayer-Anderson 2020).

*Data quality issues in Veneto*

The Veneto setting was excluded from our analyses due to inconsistencies in the cannabis data in this setting. The team collecting the data discovered several coding errors (e.g., several never users having an age at first start) which could not be clarified.

**Supplement 2: Further information on cannabis use in controls per setting, missing data, and multiple imputation by chained equations**

We included 1,335 controls in our study from 14 settings, of whom 1,304 (97.7%) had complete data on daily and high potency cannabis use. Despite the high proportion of complete data, setting-level missingness on daily cannabis use ranged from 0.0% in several settings to 23.1% in Oviedo, while setting-level missingness for high potency cannabis use ranged from 0.0% in Madrid to 35.9% in Oviedo (Supplemental Table 2i).

To minimize potential issues arising from missing cannabis use data in each setting, we performed multiple imputation by chained equations (MICE) on the control sample, using a comprehensive set of auxiliary variables (Supplemental Table 2ii) to aid imputation and add credence to the assumption that the missing data patterns were at least Missing At Random (MAR). These variables included setting, current cannabis use, age first used cannabis, number and type of other drugs used, sex, age, ethnicity, migrant status, language fluency, income, highest education, years in education, and current living arrangements. We ran 30 imputation sets following a burn-in discarding the first 10 imputation sets. Models were fitted with appropriate distributional assumptions for the exposure (binary, i.e. logistic regression) and auxiliary (linear, logistic, ordinal logistic and multinomial logistic regression) variables. Following MICE, we applied post-stratification weights to the imputed datasets to estimate the weighted proportion of daily and high potency cannabis use in each setting, based on the imputed dataset, and having taken into account differences in the control sample to the population at-risk in each setting by age group, sex and ethnicity (see Supplement 3 for details of the post-stratification weighting procedure).

Following post-stratification weighting, the prevalence of daily and high potency cannabis use in each setting was very similar in the multiply imputed dataset to the prevalence estimates derived from the “complete case” sample of controls (i.e. those with non-missing cannabis use data) (Supplemental Table 2ii). Given the low level of missing data on daily cannabis use, prevalence estimates between the imputed and complete case samples were nearly identical. There were a few larger differences between these datasets for high potency cannabis use given a higher proportion of missing data on this variable, but these differences were – overall – minor, and did not alter the range of high potency cannabis use across settings. We used the multiply imputed, post-stratified weighted estimates of the prevalence of daily and high potency cannabis use in controls as our proxies for the prevalence of cannabis use in the population at-risk in each setting in these analyses, and compared these results to those derived from several sensitivities to this methodology in Supplement 12.

*Supplemental Table 2i: Prevalence of daily and high potency cannabis use in controls, per setting, before and after multiple imputation for missing data, and levels of missingness on cannabis use in controls*

| **Setting** | **Controls**  **N (%)^a^** | **Missing daily cannabis use**  **N (%)^b^** | **Missing high potency cannabis use**  **N (%)^b^** | **% Daily cannabis (Complete data)^c^** | **% Daily cannabis (Imputed)^d^** | **% High-potency (Complete data)^c^** | **% High-potency (Imputed)^d^** |
| --- | --- | --- | --- | --- | --- | --- | --- |
| Southeast London | 230 (17.2) | 7 (3.0) | 13 (5.7) | 12.5 | 12.6 | 26.7 | 26.7 |
| Cambridgeshire | 106 (7.9) | 0 (0.0) | 11 (10.4) | 3.4 | 3.4 | 9.4 | 9.8 |
| Amsterdam | 101 (7.6) | 1 (1.0) | 10 (10.0) | 14.8 | 14.8 | 53.1 | 54.5 |
| Gouda & Voorhout | 109 (8.2) | 1 (0.9) | 15 (13.8) | 4.7 | 4.6 | 21.7 | 19.1 |
| Madrid | 38 (2.8) | 0 (0.0) | 0 (0.0) | 14.4 | 14.4 | 13.7 | 13.7 |
| Barcelona | 37 (2.8) | 0 (0.0) | 1 (2.7) | 15.5 | 15.5 | 19.4 | 19.4 |
| Valencia | 32 (2.4) | 7 (21.9) | 9 (28.2) | 9.5 | 9.5 | 9.5 | 10.4 |
| Oviedo | 39 (2.9) | 9 (23.1) | 14 (35.9) | 1.9 | 3.1 | 3.8 | 6.2 |
| Santiago | 38 (2.8) | 5 (12.8) | 12 (31.6) | 0.0 | 0.0 | 0.0 | 0.0 |
| Cuenca | 38 (2.8) | 5 (12.8) | 5 (15.8) | 1.1 | 3.0 | 3.8 | 4.5 |
| Val-de-Marne | 100 (7.5) | 0 (0.0) | 7 (7.0) | 11.9 | 11.9 | 21.1 | 20.3 |
| Bologna | 65 (4.9) | 0 (0.0) | 8 (12.4) | 1.1 | 1.1 | 9.5 | 8.7 |
| Palermo | 100 (7.5) | 0 (0.0) | 4 (4.0) | 3.4 | 3.4 | 3.9 | 3.9 |
| Ribeirão Preto | 302 (22.6) | 3 (1.0) | 6 (2.0) | 7.2 | 7.2 | 2.0 | 2.2 |

*^a^Column percentages*

*^b^Row percentages (% controls with missing cannabis data per setting)*

*^c^After post-stratification weighting for age group, sex, and ethnic/migrant group (see Supplement 3)*

*^d^Imputed using Multiple Imputation by Chained Equations (see above), with post-stratification weighting for age group, sex and ethnic/migrant group applied (see Supplement 3)*

*Supplemental Table 2ii: Auxiliary variables used in multiple imputation models of missing cannabis use data in controls*

|  | **Daily cannabis use** | | |  |
| --- | --- | --- | --- | --- |
| **Auxiliary variables^a^** | **No (N (%))** | **Yes (N (%))** | **Missing (N (%))** | **P-value^c^** |
| *Categorical variables* |  |  |  |  |
| Sex |  |  |  | 14.4 (2); p=0.001 |
| Male | 547 (86.6) | 63 (10.0) | 22 (3.5) |  |
| Female | 652 (92.8) | 35 (5.0) | 16 (2.3) |  |
| Ethnicity |  |  |  | 17.7 (12); p=0.13 |
| White | 914 (89.8) | 71 (7.0) | 33 (3.2) |  |
| Black | 104 (86.0) | 15 (12.4) | 2 (1.7) |  |
| Mixed | 109 (94.8) | 6 (5.2) | 0 (0.0) |  |
| Asian | 28 (84.5) | 2 (6.1) | 3 (9.1) |  |
| North African | 20 (87.0) | 3 (13.0) | 0 (0.0) |  |
| Other | 23 (95.8) | 1 (4.2) | 0 (0.0) |  |
| *Missing* | 1 (100.0) | 0 (0.0) | 0 (0.0) |  |
| Migrant status |  |  |  | 3.6 (2); p=0.17 |
| Non-migrant | 940 (89.0) | 84 (8.0) | 32 (3.0) |  |
| Migrant | 259 (92.8) | 14 (5.0) | 6 (2.2) |  |
| Highest education |  |  |  | 15.9 (12); p=0.19 |
| School, no qualifications | 60 (85.7) | 8 (11.4) | 2 (2.9) |  |
| School, with qualifications | 163 (91.6) | 14 (7.9) | 1 (0.6) |  |
| Tertiary education | 322 (90.4) | 23 (6.5) | 11 (3.1) |  |
| Vocational degree | 185 (84.9) | 21 (9.6) | 12 (5.5) |  |
| Undergraduate degree | 283 (92.2) | 17 (5.5) | 7 (2.3) |  |
| Postgraduate degree | 179 (90.0) | 15 (7.5) | 5 (2.5) |  |
| *Missing* | 7 (100.0) | 0 (0.0) | 0 (0.0) |  |
| Income status |  |  |  | 7.3 (6); p=0.12 |
| Below median | 542 (89.4) | 50 (8.3) | 14 (2.3) |  |
| Above median | 480 (89.6) | 34 (6.3) | 22 (4.1) |  |
| *Missing* | 177 (91.7) | 14 (7.3) | 2 (1.0) |  |
| Receipt of welfare benefits |  |  |  | 9.6 (4); p=0.05 |
| No | 830 (90.1) | 59 (6.4) | 32 (3.5) |  |
| Yes | 216 (87.5) | 26 (10.5) | 5 (2.0) |  |
| *Missing* | 153 (91.6) | 13 (7.8) | 1 (0.6) |  |
| Living status |  |  |  | 84.9 (10); p<0.001 |
| Alone^b^ | 247 (82.9) | 22 (7.4) | 29 (9.7) |  |
| With partner^b^ | 243 (90.3) | 25 (9.3) | 1 (0.4) |  |
| Parents | 325 (94.8) | 17 (5.0) | 1 (0.3) |  |
| Other family | 261 (89.7) | 27 (9.3) | 3 (1.0) |  |
| Friends | 113 (92.6) | 7 (5.7) | 2 (1.6) |  |
| *Missing* | 10 (83.3) | 0 (0.0) | 2 (16.7) |  |
| Currently using cannabis |  |  |  | 451.0 (4); p<0.001 |
| No | 1,089 (93.1) | 63 (5.4) | 18 (1.5) |  |
| Yes | 109 (72.7) | 34 (22.7) | 7 (5.7) |  |
| *Missing* | 1 (6.7) | 1 (6.7) | 13 (86.7) |  |
| Ever tried… |  |  |  |  |
| Inhalants (Yes) | 36 (66.7) | 18 (33.3) | 0 (0.0) | 56.8 (2); p<0.001 |
| Crack (Yes) | 12 (50.0) | 10 (41.2) | 2 (8.3) | 46.1 (2); p<0.001 |
| Cocaine (Yes) | 136 (71.6) | 48 (25.3) | 6 (3.2) | 105.2 (2); p<0.001 |
| Stimulants (Yes) | 119 (74.8) | 37 (23.3) | 3 (1.9) | 67.5 (2); p<0.001 |
| Sedatives (Yes) | 17 (60.7) | 11 (39.3) | 0 (0.0) | 43.3 (2); p<0.001 |
| Opioids (Yes) | 10 (52.6) | 8 (42.1) | 1 (5.3) | 35.1 (2); p<0.001 |
| Hallucinogens (Yes) | 84 (69.4) | 32 (26.5) | 5 (4.1) | 73.1 (2); p<0.001 |
| Ketamine (Yes) | 25 (67.6) | 11 (29.7) | 1 (2.7) | 28.1 (2); p<0.001 |
| Psychoactive substances (Yes) | 25 (80.7) | 6 (19.4) | 0 (0.0) | 7.4 (2); p=0.02 |
|  |  |  |  |  |
| *Continuous variables* |  |  |  |  |
| Age (in years) (median, IQR) | 33 (26-48) | 32 (25-44) | 30 (25-36) | 1.2; p=0.22 |
| Years in education (median, IQR) | 15 (12-17) | 15 (12-17) | 14 (12-17) | 0.46; p=0.64 |
| Age first tried cannabis (in years) (median, IQR) | 17 (15-20) | 16 (14-17) | 17 (15-18) | 5.3; p<0.001 |
| Language fluency^d^ (median, IQR) | 10 (10-10) | 10 (10-10) | 10 (10-10) | -0.50; p=0.62 |
| Number of drugs tried (median, IQR) | 0 (0-0) | 2 (0-3) | 0 (0-1) | -10.8; p<0.001 |

*^a^In addition to a variable to denote setting, also used during multiple imputation (see Supplemental Table 2i)*

*^b^With or without dependents*

*^c^Χ^2^ p-value for categorical variables, presented alongside the Χ^2^stattistic and degrees of freedom. Mann-Whitney U-test for continuous variables, presented alongside the U-statistic*

*^d^Language fluency rated by participants from 1 (not fluent at all) to 10 (fluent)*

**Supplement 3: Control representativeness to the population at-risk per setting and post-stratification weighting**

We assessed 1,335 controls in our 14 settings, for whom 1,297 (97.2%) had data available on daily cannabis use, 1,219 (91.3%) on high-potency cannabis use, and 1,304 (97.7%) on daily and/or high-potency cannabis use. The 1,304 controls were, on average, younger (χ^2^(2) df: 176.24, p<.001) and less often from the non-migrant/ethnic majority group (χ^2^(1) df: 27.23, p<.001) than the population at-risk (see table below), which we dealt with by applying poststratification weights.

Briefly, post-stratification is a weighting procedure that allows one to calibrate sample estimates (here: daily and high-potency cannabis use in controls) in the presence of known differences (selection biases) to the external population that the controls are purported to represent. The general principle underlying post-stratification weighting is to weight our cannabis use variables based on the ratio of the sampling fractions observed in the external population to the sampling fractions observed in controls over the available set of variables on which differences have been observed. Here, we used the known differences in our control sample to the population at-risk in each setting, by age group (18-24, 25-34, 35-64), sex (male, female), and migrant/ethnic group (majority vs. minority) (as observed in Supplemental Tables 3i and 3ii, below) to estimate post-stratification weights for our cannabis use variables. In particular, these age groups corresponded both to distinct periods of risk for psychosis, and distinct periods of higher, middling, and lower cannabis use. We applied post-stratification weights to the multiply imputed control cannabis use data (Supplement 2) in line with the post-stratification weighting procedure described in Valliant and Dever (2018). This procedure was implemented using the “survey” commands in Stata. First, we declared the weighting structure of the dataset, with controls stratified by setting, and identifying the post-stratum class to which each control belonged (i.e., the specific age, sex, and migrant/ethnic group in each setting), as well as the corresponding weighting value for that stratum (here, the estimate of the population at-risk). Second, we estimated the weighted proportion of daily and high-potency cannabis users in each setting based on the post-stratification weights. Third, we retained these estimates for fitting as exposure variables in our main incidence analyses.

*Supplemental Table 3i: Representativeness of control participants to the population at-risk in each setting, by sex and migrant/ethnic group*

|  | Sex | | | | Migrant/ethnic group | | | |
| --- | --- | --- | --- | --- | --- | --- | --- | --- |
|  | **Women** | | | | **Non-migrant/ethnic majority group** | | | |
|  | **Population^a^** | **Controls***^b^* |  |  | **Population^a^** | **Controls***^b^* |  |  |
|  | ***n* (%)** | ***n* (%)** | **χ^2^** | ***p*-value** | ***n* (%)** | ***n* (%)** | **χ^2^** | ***p*-value** |
| England |  |  |  |  |  |  |  |  |
| Southeast London | 213,472 (50.1) | 113 (50.7) | 0.03 | 0.854 | 175,706 (41.2) | 104 (46.6) | 2.72 | 0.099 |
| Cambridgeshire | 771,816 (49.7) | 56 (52.8) | 0.43 | 0.513 | 1,238,172 (79.7) | 85 (80.2) | 0.02 | 0.891 |
| The Netherlands |  |  |  |  |  |  |  |  |
| Amsterdam | 307,854 (49.6) | 53 (53.0) | 0.47 | 0.492 | 293,709 (47.3) | 56 (56.0) | 3.05 | 0.081 |
| Gouda and Voorhout | 381,795 (49.8) | 57 (52.3) | 0.27 | 0.602 | **651,786 (85.0)** | **103 (94.5)** | **7.70** | **0.006** |
| Spain |  |  |  |  |  |  |  |  |
| Madrid | 209,422 (50.5) | 20 (52.6) | 0.07 | 0.792 | 329,424 (79.4) | 28 (73.7) | 0.76 | 0.382 |
| Barcelona | 457,635 (51.8) | 21 (56.8) | 0.37 | 0.544 | 688,285 (77.9) | 28 (75.7) | 0.10 | 0.748 |
| Valencia | 183,496 (50.4) | 15 (55.6) | 0.29 | 0.591 | 299,983 (82.4) | 22 (81.5) | 0.01 | 0.904 |
| Oviedo | 235,644 (50.9) | 17 (54.8) | 0.19 | 0.664 | **428,482 (92.6)** | **25 (80.7)** | **6.50** | **0.011** |
| Santiago | 288,177 (50.1) | 18 (51.4) | 0.02 | 0.877 | 556,193 (96.7) | 34 (97.1) | FE | 0.683 |
| Cuenca | 92,379 (47.4) | 16 (47.1) | 0.00 | 0.972 | 160,723 (82.4) | 27 (79.4) | 0.21 | 0.648 |
| France |  |  |  |  |  |  |  |  |
| Val-de-Marne | 268,299 (52.5) | 53 (53.0) | 0.01 | 0.927 | **342,090 (67.0)** | **51 (51.0)** | **11.57** | **0.001** |
| Italy |  |  |  |  |  |  |  |  |
| Bologna | 475,917 (51.1) | 41 (63.1) | 3.74 | 0.053 | **727,700 (78.1)** | **60 (92.3)** | **7.67** | **0.006** |
| Palermo | 813,878 (51.0) | 51 (51.0) | 0.00 | 0.995 | 1,493,856 (93.7) | 92 (92.0) | 0.47 | 0.494 |
| Brazil |  |  |  |  |  |  |  |  |
| Ribeirão Preto | 1,332,577 (50.6) | 157 (52.5) | 0.42 | 0.517 | 1,745,638 (66.3) | 203 (67.9) | 0.33 | 0.568 |
| Total | 6,032,361 (50.6) | 688 (52.8) | 2.55 | 0.110 | **9,131,747 (76.5)** | **918 (70.4)** | **27.23** | **<0.001** |

*Bold: p<0.05 statistically significant; FE: Fisher’s Exact test performed instead of χ^2^ due to cells <5*

*^a^Total number of population at-risk: 11,933,250*

*^b^Total number of controls: 1,302 (age; 2 controls with missing age), 1,304 (sex, migrant/ethnic group)*

*Supplemental Table 3ii: Representativeness of control participants to the population at-risk in each setting, by age group*

|  | Age | | Age | | Age | |  |  |
| --- | --- | --- | --- | --- | --- | --- | --- | --- |
|  | **18-24 years** | | **25-34 years** | | **35-64 years** | |  |  |
|  | **Population^a^** | **Controls***^b^* | **Population^a^** | **Controls***^b^* | **Population^a^** | **Controls***^b^* |  |  |
|  | ***n* (%)** | ***n* (%)** | ***n* (%)** | ***n* (%)** | ***n* (%)** | ***n* (%)** | **χ^2^** | ***p*-value** |
| England |  |  |  |  |  |  |  |  |
| Southeast London | **66,551 (15.6)** | **47 (21.1)** | **148,162 (34.7)** | **101 (45.3)** | **211,740 (49.7)** | **75 (33.6)** | **22.93** | **<0.001** |
| Cambridgeshire | 237,738 (15.3) | 14 (13.2) | 345,003 (22.2) | 23 (21.7) | 971,682 (62.5) | 69 (65.1) | 0.43 | 0.808 |
| The Netherlands |  |  |  |  |  |  |  |  |
| Amsterdam | **96,909 (15.6)** | **30 (30.0)** | **159,660 (25.7)** | **22 (22.0)** | **364,572 (58.7)** | **48 (48.0)** | **15.77** | **<0.001** |
| Gouda and Voorhout | 104,808 (13.7) | 23 (21.1) | 138,114 (18.0) | 15 (13.8) | 523,848 (68.3) | 71 (65.1) | 5.66 | 0.059 |
| Spain |  |  |  |  |  |  |  |  |
| Madrid | **47,660 (11.5)** | **13 (43.2)** | **102,013 (24.6)** | **4 (10.5)** | **265,115 (63.9)** | **21 (55.3)** | **FE** | **<0.001** |
| Barcelona | 92,800 (10.5) | 8 (21.6) | 218,864 (24.8) | 6 (16.2) | 572,229 (64.7) | 23 (62.2) | 5.49 | 0.064 |
| Valencia | 41,491 (11.4) | 1 (3.7) | 83,749 (23.0) | 6 (22.2) | 238,954 (65.6) | 20 (74.1) | FE | 0.513 |
| Oviedo | **43,502 (9.4)** | **3 (9.7)** | **98,856 (21.4)** | **15 (48.4)** | **320,266 (69.2)** | **13 (41.9)** | **FE** | **0.002** |
| Santiago | 61,468 (10.7) | 1 (2.9) | 127,665 (22.2) | 8 (23.5) | 385,807 (67.1) | 25 (73.5) | FE | 0.396 |
| Cuenca | 25,647 (13.1) | 7 (20.6) | 43,026 (22.1) | 7 (20.6) | 126,402 (64.8) | 20 (58.8) | 1.65 | 0.438 |
| France |  |  |  |  |  |  |  |  |
| Val-de-Marne | 80,692 (15.8) | 22 (22.0) | 118,076 (23.1) | 28 (28.0) | 311,865 (61.1) | 50 (50.0) | 5.47 | 0.065 |
| Italy |  |  |  |  |  |  |  |  |
| Bologna | **82,417 (8.8)** | **20 (30.8)** | **190,570 (20.5)** | **23 (35.4)** | **658,759 (70.7)** | **22 (33.9)** | **54.88** | **<0.001** |
| Palermo | **222,314 (13.9)** | **34 (34.0)** | **319,158 (20.0)** | **30 (30.0)** | **1,053,410 (66.0)** | **36 (36.0)** | **47.52** | **<0.001** |
| Brazil |  |  |  |  |  |  |  |  |
| Ribeirão Preto | **499,242 (19.0)** | **54 (18.1)** | **719,871 (27.4)** | **160 (53.7)** | **1,412,574 (53.7)** | **84 (28.2)** | **111.73** | **<0.001** |
| Total | **1,703,240 (14.3)** | **277 (21.3)** | **2,812,788 (23.6)** | **448 (34.4)** | **7,417,224 (62.2)** | **577 (44.3)** | **176.24** | **<0.001** |

*Bold: p<0.05 statistically significant; FE: Fisher’s Exact test performed instead of χ^2^ due to cells <5*

*^a^Total number of population at-risk: 11,933,250*

*^b^Total number of controls: 1,302 (age; 2 controls with missing age), 1,304 (sex, migrant/ethnic group)*

**Supplement 4: Additional information on the statistical analyses**

*Multilevel data structure*

We applied a multilevel structure where participants (level 1) were nested within settings (level 2) in the regression analyses. We checked evidence of clustering by running a null model (without covariates) for each outcome. This provided us with an estimate of the between-setting variance ($\sigma_{u}^{2}$) in incidence rates of any given outcome, and the corresponding standard error (SE). We also reported the Wald test for null and multivariable models to determine whether this between-setting variance was statistically significant from zero (no residual variance at the setting level).

*Negative binomial vs Poisson regression*

Outcome data in 4 out of the 6 (3 outcomes; null and fully adjusted) models were over-dispersed, i.e., the conditional variance of the outcome variable exceeded the conditional mean, justifying the use of negative binomial regression models across all outcomes for comparability in this study (Hilbe 2011). The only exception to this was for affective psychotic disorders, consistent with previous evidence that affective psychotic disorders show weak or no associations with area-level variance (March 2008). Nonetheless, for consistency, all models were run using negative binomial regression modeling.

**Supplement 5:** **Sample characteristics for non-affective and affective psychotic disorders**

*Supplemental Table 5i: Detailed sample characteristics of incidence cases, by diagnostic category*

|  | | **Non-affective psychotic disorders** | | | |  | **Affective psychotic disorders** | | | |
| --- | --- | --- | --- | --- | --- | --- | --- | --- | --- | --- |
| **Setting** | | **Cases,**  **N (%)^a^** | **Women,**  **N (%)^b^** | **Age at first contact <35 (years),**  **N (%)^b^** | **Migrant/ ethnic minorities, N (%)^b^** |  | **Cases,**  **N (%)^a^** | **Women,**  **N (%)^c^** | **Age at first contact <35 (years),**  **N (%)^c^** | **Migrant/ ethnic minorities, N (%)^c^** |
|  | Southeast London | 245 (93.5) | 110 (44.9) | 135 (55.1) | 189 (77.1) |  | 17 (6.5) | 11 (64.7) | 9 (52.9) | 12 (70.6) |
|  | Cambridgeshire | 173 (65.0) | 69 (39.9) | 132 (76.3) | 73 (42.2) |  | 90 (33.8) | 46 (51.1) | 55 (61.1) | 29 (32.2) |
|  | Amsterdam | 258 (88.1) | 87 (33.7) | 152 (58.9) | 187 (72.5) |  | 34 (11.6) | 16 (47.1) | 22 (64.7) | 17 (50.0) |
|  | Gouda and Voorhout | 122 (73.5) | 42 (34.4) | 93 (76.2) | 34 (27.9) |  | 39 (23.5) | 21 (53.8) | 16 (41.0) | 5 (12.8) |
|  | Madrid | 71 (80.7) | 21 (29.6) | 45 (63.4) | 11 (15.5) |  | 12 (13.6) | 6 (50.0) | 4 (33.3) | 1 (8.3) |
|  | Barcelona | 95 (88.0) | 40 (42.1) | 69 (72.6) | 26 (27.4) |  | 9 (8.3) | 4 (44.4) | 6 (66.7) | 0 (0.0) |
|  | Valencia | 51 (86.4) | 22 (43.1) | 31 (60.8) | 8 (15.7) |  | 6 (10.2) | 4 (66.7) | 5 (83.3) | 1 (16.7) |
|  | Oviedo | 60 (73.2) | 31 (51.7) | 33 (55.0) | 13 (21.7) |  | 18 (22.0) | 10 (55.6) | 8 (44.4) | 1 (5.6) |
|  | Santiago | 28 (77.8) | 13 (46.4) | 12 (42.9) | 1 (3.6) |  | 5 (13.9) | 0 (0.0) | 4 (80.0) | 0 (0.0) |
|  | Cuenca | 26 (96.3) | 6 (23.1) | 19 (73.1) | 6 (23.1) |  | 0 (0.0) | NA | NA | NA |
|  | Val-de-Marne | 134 (63.8) | 56 (41.8) | 80 (59.7) | 40 (29.9) |  | 76 (36.2) | 46 (60.5) | 44 (57.9) | 27 (35.5) |
|  | Bologna | 124 (75.2) | 59 (47.6) | 78 (62.9) | 39 (31.5) |  | 41 (24.8) | 20 (48.8) | 29 (70.7) | 10 (24.4) |
|  | Palermo | 151 (84.4) | 66 (43.7) | 96 (63.6) | 21 (13.9) |  | 27 (15.1) | 12 (44.4) | 17 (63.0) | 1 (3.7) |
|  | Ribeirão Preto | 281 (54.1) | 110 (39.1) | 176 (62.6) | 128 (45.6) |  | 237 (45.7) | 139 (58.6) | 132 (55.7) | 113 (47.7) |
| Total | | 1,819 (73.9) | 732 (40.2) | 1,151 (63.3) | 776 (42.7) |  | 611 (24.8) | 335 (54.8) | 351 (57.4) | 217 (35.5) |

*FEP: first episode psychosis, IQR: interquartile range, NA: not applicable*

*^a^Of all cases for each setting*

*^b^Of all non-affective psychotic disorders for each setting*

*^c^Of all affective psychotic disorders for each setting*

**Supplement 6: Overall sample characteristics of incidence cases**

*Supplemental Table 6i: Sample characteristics of incidence cases by sex, age, migrant/ethnic group*

|  | | **Incidence cases** | | **Female cases** | | **Migrant or ethnic minority group** | | **Non-affective psychosis cases** | | **Affective psychosis cases** | |
| --- | --- | --- | --- | --- | --- | --- | --- | --- | --- | --- | --- |
|  |  | ***n*** | **%^a^** | ***n*** | **%^b^** | ***n*** | **%^b^** | ***n*** | **%^b^** | ***n*** | **%^b^** |
| Age (years) | |  |  |  |  |  |  |  |  |  |  |
|  | 18-24 | 749 | 30.4 | 238 | 31.8 | 305 | 40.7 | 566 | 75.6 | 173 | 23.1 |
|  | 25-29 | 427 | 17.4 | 167 | 39.1 | 180 | 42.2 | 335 | 78.5 | 88 | 20.6 |
|  | 30-34 | 343 | 13.9 | 159 | 46.4 | 148 | 43.1 | 250 | 72.9 | 90 | 26.2 |
|  | 35-39 | 262 | 10.7 | 133 | 50.8 | 112 | 42.7 | 206 | 78.6 | 52 | 19.8 |
|  | 40-44 | 211 | 8.6 | 103 | 48.8 | 87 | 41.2 | 160 | 75.8 | 49 | 23.2 |
|  | 45-49 | 193 | 7.8 | 114 | 59.1 | 75 | 38.9 | 123 | 63.7 | 65 | 33.7 |
|  | 50-55 | 136 | 5.5 | 86 | 63.2 | 48 | 35.3 | 89 | 65.4 | 45 | 33.1 |
|  | 55-59 | 89 | 3.6 | 50 | 56.2 | 26 | 29.2 | 59 | 66.3 | 30 | 33.7 |
|  | 60-64 | 50 | 2.0 | 30 | 60.0 | 18 | 36.0 | 31 | 62.0 | 19 | 38.0 |
| Sex | |  |  |  |  |  |  |  |  |  |  |
|  | Women | 1,080 | 43.9 | 1,080 | 100.0 | 445 | 41.2 | 732 | 67.8 | 335 | 31.0 |
|  | Men | 1,380 | 56.1 | - | - | 554 | 40.1 | 1087 | 78.8 | 276 | 20.0 |
| Migrant/ethnic group | |  |  |  |  |  |  |  |  |  |  |
|  | Migrant or ethnic minority group | 999 | 40.6 | 445 | 44.5 | 999 | 100.0 | 776 | 77.7 | 217 | 21.7 |
|  | Non-migrant or ethnic majority group | 1,461 | 59.4 | 635 | 43.5 | - | - | 1043 | 71.4 | 394 | 27.0 |
| Total | | 2,460 | 100.0 | 1,080 | 43.9 | 999 | 40.6 | 1,819 | 73.9 | 611 | 24.8 |
| Crude incidence (95% CI)^c^ | | 20.6 (19.8-21.4) | | 17.9 (16.9-19.0) | | 36.3 (34.1-38.7) | | 15.2 (14.6-16.0) | | 5.1 (4.7-5.5) | |

*^a^Column percentages*

*^b^Row percentages*

*^c^Per 100,000 person-years*

*Supplemental Figure 6i: Age distribution of incidence cases of FEP, including cumulative incidence, stratified by sex*

**Supplement 7: Overall sample characteristics for the person-years at-risk**

*Supplemental Table 7i: Sample characteristics of person-years at risk by sex, age, migrant/ethnic group*

| **Setting** | | **Person years at-risk** | **Women, N (%)** | **Aged under 35 years, N (%)** | **Migrant/ethnic minorities, N (%)** |
| --- | --- | --- | --- | --- | --- |
| England | | | | | |
|  | Southeast London | 426,453 | 213,472 (50.1) | 214,713 (50.3) | 250,747 (58.8) |
|  | Cambridgeshire | 1,554,423 | 771,816 (49.7) | 582,741 (37.5) | 316,251 (20.3) |
| The Netherlands | | | | | |
|  | Amsterdam | 621,141 | 307,854 (49.6) | 256,569 (41.3) | 327,432 (52.7) |
|  | Gouda and Voorhout | 766,770 | 381,795 (49.8) | 242,922 (31.7) | 114,984 (15.0) |
| Spain | | | | | |
|  | Madrid | 414,787.4 | 209,420.5 (50.5) | 149,672.1 (36.1) | 85,362.2 (20.6) |
|  | Barcelona | 883,893 | 457,634.7 (51.8) | 311,664.3 (35.3) | 195,609.7 (22.1) |
|  | Valencia | 364,193.5 | 183,495.7 (50.4) | 125,239.8 (34.4) | 64,210.3 (17.6) |
|  | Oviedo | 462,624.6 | 235,644.3 (50.9) | 142,358.2 (30.8) | 34,141.3 (7.4) |
|  | Santiago | 574,940.7 | 288,173.7 (50.1) | 189,133.5 (32.9) | 18,749.1 (3.3) |
|  | Cuenca | 195,074.9 | 92378.5 (47.4) | 68,673.3 (35.2) | 34,350.9 (17.6) |
| France | | | | | |
|  | Val-de-Marne | 510,633.7 | 268,300.2 (52.5) | 198,768.3 (38.9) | 147,437.8 (28.9) |
| Italy | | | | | |
|  | Bologna | 931,746 | 475,917 (51.1) | 272,987 (29.3) | 173,203 (18.6) |
|  | Palermo | 1,594,883 | 813,880.5 (51.0) | 541,472.5 (34.0) | 101,026.1 (6.3) |
| Brazil | | | | | |
|  | Ribeirão Preto | 2,631,687 | 1,332,576 (50.6) | 1,219,113 (46.3) | 886,050 (33.7) |
| Total | | 11,933,250.8 | 6,032,358.1 (50.6) | 4,516,027.0 (37.8) | 2,749,554.4 (23.0) |

**Supplement 8: Comparison of the incidence cases with the person-years at-risk**

*Supplemental Table 8i: Comparison of incidence cases with person-years at risk, by age, sex migrant/ethnic group*

|  | | **Incidence cases** | | **Person-years at-risk** | |  |  |
| --- | --- | --- | --- | --- | --- | --- | --- |
|  |  | **n** | **%** | **n** | **%** | **χ^2^** | ***p-*value** |
| Age (years) | |  |  |  |  | 825.16 | <0.001 |
|  | 18-24 | 749 | 30.4 | 1,703,239.7 | 14.3 |  |  |
|  | 25-29 | 427 | 17.4 | 1,388,483.5 | 11.6 |  |  |
|  | 30-34 | 343 | 13.9 | 1,424,304.0 | 11.9 |  |  |
|  | 35-39 | 262 | 10.7 | 1,386,089.9 | 11.6 |  |  |
|  | 40-44 | 211 | 8.6 | 1,385,247.0 | 11.6 |  |  |
|  | 45-49 | 193 | 7.8 | 1,356,310.9 | 11.4 |  |  |
|  | 50-55 | 136 | 5.5 | 1,238,503.3 | 10.4 |  |  |
|  | 55-59 | 89 | 3.6 | 1,075,628.5 | 9.0 |  |  |
|  | 60-64 | 50 | 2.0 | 975,444.0 | 8.2 |  |  |
| Sex | |  |  |  |  | 43.49 | <0.001 |
|  | Women | 1,080 | 43.9 | 6,032,358.1 | 50.6 |  |  |
|  | Men | 1,380 | 56.1 | 5,900,892.7 | 49.4 |  |  |
| Migrant/ethnic group | |  |  |  |  | 428.07 | <0.001 |
|  | Migrant or ethnic minority group | 999 | 40.6 | 2,749,554.4 | 23.0 |  |  |
|  | Non-migrant or ethnic majority group | 1,461 | 59.4 | 9,183,696.4 | 77.0 |  |  |
| Total |  | 2,460 | 100.0 | 11,933,250.8 | 100.0 | - | - |

**Supplement 9: Descriptive statistics of setting-level variables**

*Supplemental Table 9i: Descriptive statistics of setting-level variables*

| **Setting-level variables** | **Mean** | **95% CI** | **SD** | **Median** | **IQR** |
| --- | --- | --- | --- | --- | --- |
| Daily cannabis (%)^a^ | 7.2 | 5.7-8.7 | - | 5.9 | 3.1-12.6 |
| High-potency cannabis (%)^a^ | 12.2 | 10.4-14.0 | - | 10.1 | 4.5-19.5 |
| Owner-occupancy (%)^b^ | 67.9 | - | 15.2 | 72.9 | 58.7-79.9 |
| Unemployment (%)^b^ | 9.0 | - | 5.5 | 6.8 | 4.2-13.8 |
| Population density (people per km^2^) | 4169.8 | - | 4486.8 | 3960.6 | 145.2-4997.2 |

*^a^prevalence in controls following multiple imputation, with poststratification weights applied*

*^b^prevalence in population*

**Supplement 10: Correlation matrices and scatterplots of setting-level variables**

Below, we report Spearman correlation coefficients between the setting-level variables in Supplemental Table 10i, and provide scatterplots of statistically significant associations in Supplemental Figure 10i.

*Supplemental Table 10i: Correlation matrix between setting-level variables in 14 EU-GEI settings*

|  | Daily cannabis^a^ | High-potency cannabis^a^ | Owner-occupancy | Unemployment | Population density (people per km^2^) |
| --- | --- | --- | --- | --- | --- |
| Daily cannabis^a^ | 1 |  |  |  |  |
| High-potency cannabis^a^ | 0.79* | 1 |  |  |  |
| Owner-occupancy | -0.38 | -0.67* | 1 |  |  |
| Unemployment | -0.03 | -0.28 | 0.70* | 1 |  |
| Population density (people per km^2^) | 0.78* | 0.71* | -0.31 | 0.09 | 1 |

*^*^p<0.05 statistically significant*

*^a^Prevalence of daily- or high-potency cannabis use in controls following multiple imputation, with poststratification weights applied*

*
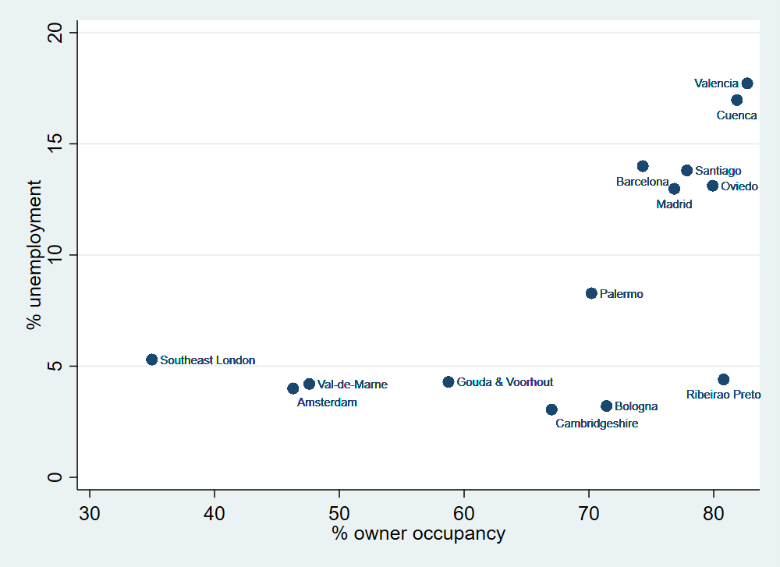

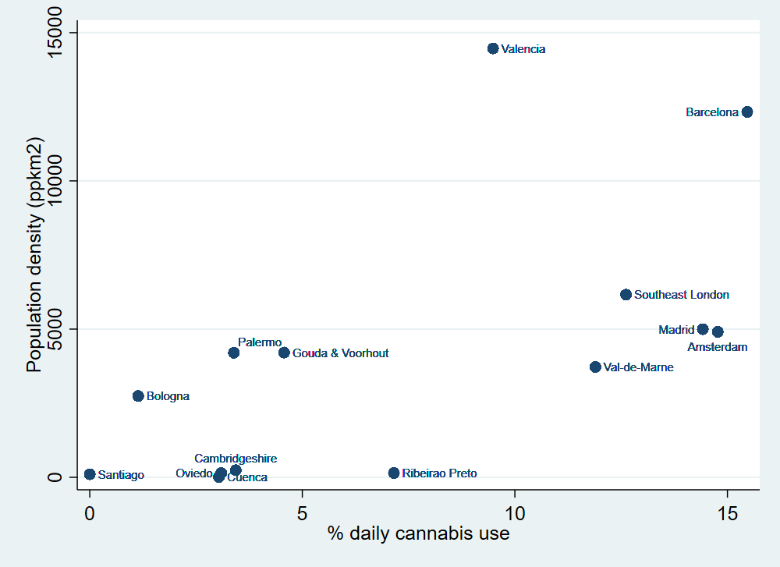

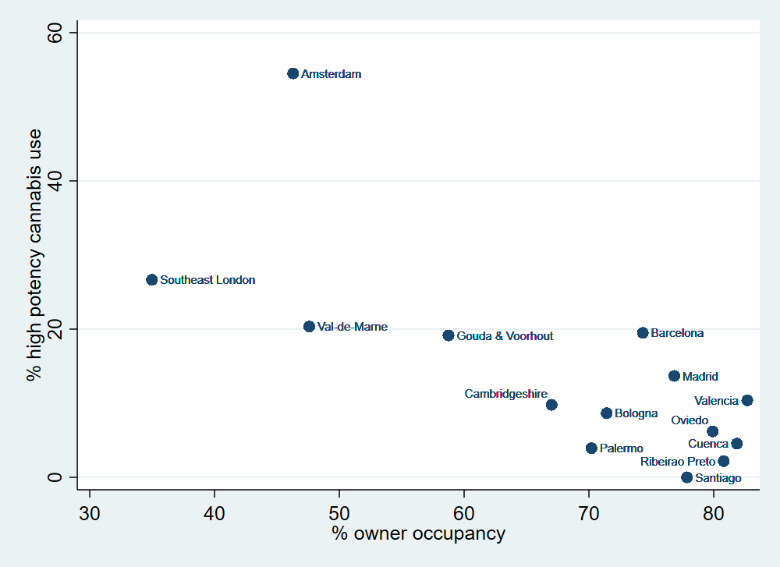

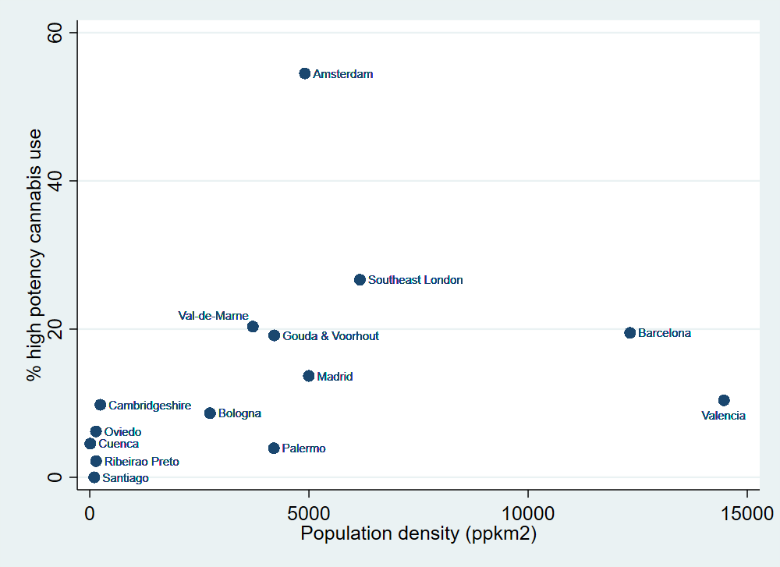

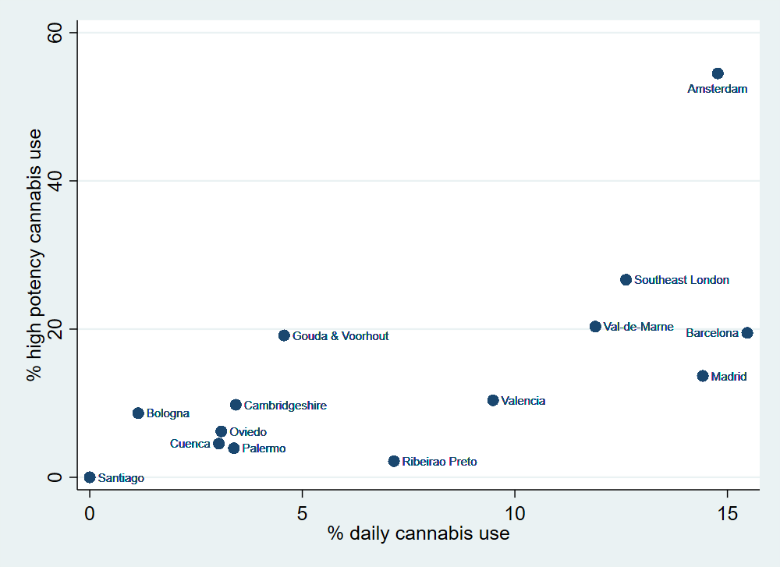
Figure 10i: Selected scatterplots of setting-level variables with statistically significant correlations*

**Legend**: Scatterplots of statistically significant correlations (Supplemental Table 10i) between setting-level variables across 14 settings in the EU-GEI study: A. *r*=0.79; B. *r*=0.78; C. *r*=-0.67; D. *r*=0.71; E. *r*=0.70.

**A.**

**B.**

**C.**

**D.**

**E.**

**Supplement 11: Full model parameter estimates from negative binomial multilevel modelling**

Null multilevel negative binomial random intercepts regression models provided evidence of substantial variation in incidence rates for all outcomes (i.e., all FEP: $\sigma_{u}^{2}$=0.26, standard error (SE)=0.11, p=0.02; non-affective psychotic disorders: $\sigma_{u}^{2}$=0.27, SE=0.12, p=0.02; affective psychotic disorders: $\sigma_{u}^{2}$=0.73, SE=0.34, p=0.03). These effects were attenuated in fully adjusted models towards the null (i.e., all FEP: $\sigma_{u}^{2}$=0.04, SE=0.02, p=0.05; non-affective psychotic disorders: $\sigma_{u}^{2}$=0.03, SE=0.02, p=0.09; affective psychotic disorders: $\sigma_{u}^{2}$=0.14, SE=0.08, p=0.10).

*Supplemental Table 11i: Univariable and multivariable random intercepts negative binomial regression of the association between individual-level covariates and all FEP, non-affective psychotic disorders, and affective psychotic disorders incidence*

|  |  | **All FEP** | | **Non-affective psychotic disorders** | | **Affective psychotic disorders** | |
| --- | --- | --- | --- | --- | --- | --- | --- |
| **Independent variable** | | **Univariable IRR (95% CI)** | **Multivariable IRR (95% CI)^a^** | **Univariable IRR (95% CI)** | **Multivariable IRR (95% CI)^a^** | **Univariable IRR (95% CI)** | **Multivariable IRR (95% CI)^a^** |
| Age at first contact (years) | 18-24  25-29  30-34  35-39  40-44  45-49  50-54  55-59  60-64 | Ref  **0.56 (0.44-0.71)**  **0.37 (0.28-0.47)**  **0.27 (0.21-0.36)**  **0.22 (0.17-0.29)**  **0.17 (0.12-0.23)**  **0.12 (0.08-0.17)**  **0.11 (0.08-0.16)**  **0.06 (0.04-0.11)** | Ref  **0.57 (0.47-0.70)**  **0.38 (0.31-0.48)**  **0.29 (0.23-0.36)**  **0.24 (0.19-0.31)**  **0.19 (0.14-0.25)**  **0.13 (0.10-0.19)**  **0.12 (0.09-0.18)**  **0.07 (0.05-0.12)** | Ref  **0.57 (0.43-0.76)**  **0.35 (0.26-0.47)**  **0.28 (0.20-0.39)**  **0.21 (0.15-0.29)**  **0.14 (0.10-0.21)**  **0.09 (0.06-0.14)**  **0.08 (0.05-0.13)**  **0.04 (0.02-0.03)** | Ref  **0.59 (0.46-0.74)**  **0.37 (0.28-0.48)**  **0.29 (0.22-0.39)**  **0.23 (0.17-0.31)**  **0.16 (0.11-0.22)**  **0.11 (0.07-0.16)**  **0.09 (0.06-0.14)**  **0.05 (0.03-0.09)** | Ref  **0.47 (0.30-0.74)**  **0.45 (0.29-0.71)**  **0.24 (0.14-0.42)**  **0.28 (0.16-0.47)**  **0.28 (0.16-0.48)**  **0.27 (0.16-0.48)**  **0.28 (0.15-0.49)**  **0.18 (0.09-0.37)** | Ref  **0.49 (0.33-0.75)**  **0.45 (0.30-0.69)**  **0.25 (0.15-0.43)**  **0.30 (0.18-0.49)**  **0.30 (0.18-0.50)**  **0.29 (0.17-0.50)**  **0.30 (0.17-0.53)**  **0.19 (0.10-0.40)** |
| Female sex |  | **0.41 (0.32-0.53)** | **0.43 (0.35-0.53)** | **0.35 (0.26-0.47)** | **0.37 (0.28-0.47)** | 0.35 (0.26-0.47) | 0.79 (0.55-1.11) |
| Age at first contact (years) x female sex | 18-24 x female  25-29 x female  30-34 x female  35-39 x female  40-44 x female  45-49 x female  50-54 x female  55-59 x female  60-64 x female | Ref  0.80 (0.60-1.06)  0.76 (0.57-1.00)  **0.68 (0.51-0.91)**  **0.53 (0.39-0.72)**  **0.60 (0.44-0.81)**  **0.49 (0.35-0.67)**  **0.32 (0.22-0.46)**  **0.22 (0.14-0.33)** | Ref  **0.82 (0.67-0.99)**  **0.78 (0.64-0.96)**  **0.71 (0.57-0.88)**  **0.55 (0.44-0.70)**  **0.63 (0.50-0.78)**  **0.52 (0.41-0.66)**  **0.35 (0.26-0.48)**  **0.24 (0.16-0.35)** | Ref  0.82 (0.58-1.16)  0.73 (0.52-1.03)  0.75 (0.53-1.06)  **0.56 (0.39-0.81)**  **0.51 (0.35-0.75)**  **0.48 (0.32-0.70)**  **0.34 (0.22-0.52)**  **0.21 (0.12-0.35)** | Ref  0.83 (0.65-1.05)  **0.74 (0.58-0.95)**  **0.77 (0.59-0.98)**  **0.59 (0.45-0.78)**  **0.55 (0.41-0.73)**  **0.51 (0.38-0.69)**  **0.38 (0.26-0.54)**  **0.24 (0.15-0.38)** | Ref  0.72 (0.46-1.11)  0.87 (0.57-1.32)  **0.51 (0.32-0.83)**  **0.44 (0.27-0.73)**  0.76 (0.49-1.17)  **0.49 (0.30-0.81)**  **0.29 (0.16-0.54)**  **0.24 (0.12-0.48)** | Ref  0.78 (0.54-1.11)  0.91 (0.65-1.29)  **0.59 (0.39-0.88)**  **0.48 (0.31-0.74)**  0.80 (0.55-1.15)  **0.54 (0.34-0.83)**  **0.32 (0.18-0.57)**  **0.26 (0.13-0.50)** |
| Migrant/ethnic minority groups |  | **1.66 (1.42-1.95)** | **1.60 (1.45-1.78)** | **1.78 (1.48-2.14)** | **1.70 (1.50-1.93)** | **1.36 (1.09-1.71)** | **1.36 (1.12-1.64)** |

*CI: confidence interval, FEP: first episode psychosis, IRR: incidence rate ratio, NA: not applicable, ref: reference category*

*Bold: p<0.05 statistically significant*

*^a^Adjusted for daily cannabis use, high-potency cannabis use, owner-occupancy, unemployment, population density, and all other variables in the table*

**Supplement 12: Sensitivity analyses**

In Supplemental Table 12i, we compared our main results (from Table 2) against those from three sensitivity analyses. These results all show the association between the incidence of selected psychotic disorders against setting-level variables, in univariable and multivariable models. Our main regression results (MI in Supplemental Table 12i) are derived following the use of multiple imputation by chained equations (MICE) to estimate the prevalence of daily and high potency cannabis use in controls in each setting, accounting for missing data in the control sample (Supplement 2).

In our first sensitivity, our main results are compared against results from regression models when we substitute the prevalence of daily and high potency cannabis use in controls using estimates derived from controls with complete data only (CC in Supplemental Table 12i; see Supplement 2 for missing cannabis data patterns by setting). All univariable and multivariable results are nearly identical to each other, suggesting that the presence of missing cannabis data did not introduce substantial bias into our results, and that missing data patterns were at least missing at random, and could be validly imputed using other observed characteristics (Supplement 2).

Our second sensitivity compares our main (MI) results to the same results, using multiply imputed prevalence estimates for daily and high potency cannabis use, but restricted to 10 of the 11 settings included in the previous publication by Di Forti et al (2019), which reported strong correlations between the incidence of all FEP and the prevalence of daily (*r*=0.80; p=0.01) and high potency (*r*=0.70; p=0.03) cannabis use in controls. We excluded one of the eleven settings included by Di Forti et al (2019), because of entirely missing data on migration/ethnicity, which prevented the estimation of incidence rates by this characteristic. Di Forti et al (2019) excluded 6 of 17 settings because of data quality issues: Maison Blanche (Paris) due to no controls, Veneto (Italy) due to low quality cannabis use data, and Valencia (Spain), Oviedo (Spain), Cuenca (Spain), Santiago (Spain) due to greater than 10% missing data on cannabis or other confounding variables. We compared our main results in 14 settings to results following the same regression analyses (adjusting for setting-level covariates, using cannabis use prevalence estimates following multiple imputation and post-stratification weighting) restricted to the subset of 10 of the 11 settings included by Di Forti et al (2019). Our main results (MI) were very similar to those obtained when restricted to this subset of 10 settings (MI_10_; Supplemental Table 12i), with the exception of the association between prevalence of daily cannabis use in controls and incidence of all FEP which became statistically significant when restricted to the 10-setting analysis (IRR: 1.31; 95%CI: 1.10-1.55).

Our final sensitivity analysis replicated the correlational results reported by Di Forti et al (2019) (described above) as closely as possible via regression modelling. As for the previous sensitivity, we restricted these analyses to the 10 settings used by Di Forti et al (2019) with complete incidence data, with multivariable regression models adjusted for age group, sex, their interaction, and broad ethnic/migrant group, but not other setting-level variables. As per Di Forti et al (2019), prevalence estimates of daily and high potency cannabis use in controls were based on complete data without post-stratification weighting applied (Supplemental Table 12ii). In contrast to our main results (MI; Supplemental Table 12i), regression modelling results from these analyses (CC_10_; Supplemental Table 12i) found strong associations between greater prevalence of *daily* cannabis use in controls and the incidence of all FEP (IRR: 1.46; 95%CI: 1.17-1.82) and non-affective psychotic disorders (IRR: 1.58; 95%CI: 1.24-2.01), after adjustment for age, sex and ethnic/migrant group. This sensitivity analysis also found strong associations between the prevalence of *high potency* cannabis use in controls and the incidence of all FEP (IRR: 1.40; 95%CI: 1.14-1.70) and non-affective psychotic disorders (IRR: 1.49; 95%CI: 1.24-1.80), after adjustment for age, sex and ethnic/migrant group. No statistically significant associations were found with respect to the incidence of affective psychotic disorders. These results contrast with our main results (MI) and other sensitivity analyses (CC, MI_10_), which we interpret as arising when analyses do not account for potential confounding by other covariates, missing data issues and post-stratification weighting.

*Supplemental Table 12i: Univariable and multivariable associations between incidence of psychotic disorders and setting-level variables in our main analysis (MI) and three sensitivity analyses (CC, MI_10_, CC_10_)*

|  |  | **All FEP** | | **Non-affective psychotic disorders** | | **Affective psychotic disorders** | |
| --- | --- | --- | --- | --- | --- | --- | --- |
| **Setting-level variable^a^** | **Analysis^b^** | **Univariable IRR**  **(95% CI)** | **Multivariable IRR (95% CI)^c^** | **Univariable IRR (95% CI)** | **Multivariable IRR (95% CI)^c^** | **Univariable IRR (95% CI)** | **Multivariable IRR**  **(95% CI)^c^** |
| Owner-occupancy | MI | **0.65 (0.56-0.75)** | **0.76 (0.61-0.95)** | **0.63 (0.55-0.72)** | **0.68 (0.55-0.83)** | **0.65 (0.43-0.99)** | 1.07 (0.70-1.64) |
|  | CC | **0.65 (0.56-0.75)** | **0.75 (0.60-0.95)** | **0.63 (0.55-0.72)** | **0.68 (0.56-0.83)** | **0.65 (0.43-0.99)** | 1.06 (0.68-1.64) |
|  | MI_10_ | **0.65 (0.58-0.74)** | **0.73 (0.63-0.84)** | **0.61 (0.54-0.68)** | **0.63 (0.52-0.75)** | 0.79 (0.51-1.24) | 1.03 (0.72-1.48) |
|  | CC_10_ ^e^ | **-** | **-** | **-** | **-** | - | - |
| Daily cannabis | MI | **1.34 (1.04-1.73)** | 1.19 (0.96-1.48) | **1.38 (1.07-1.78)** | 1.06 (0.86-1.30) | 1.18 (0.72-1.92) | **1.53 (1.02-2.31)** |
|  | CC | **1.34 (1.04-1.72)** | 1.18 (0.94-1.47) | **1.37 (1.06-1.77)** | 1.04 (0.85-1.28) | 1.22 (0.74-2.00) | 1.52 (1.00-2.32)^d^ |
|  | MI_10_ | 1.21 (0.90-1.61) | **1.31 (1.10-1.55)** | 1.28 (0.94-1.74) | 1.04 (0.84-1.29) | 0.90 (0.56-1.45) | **1.97 (1.33-2.92)** |
|  | CC_10_^e^ | **1.47 (1.15-1.88)** | **1.46 (1.17-1.82)** | **1.55 (1.20-2.01)** | **1.58 (1.24-2.01)** | 1.12 (0.66-1.88) | 1.06 (0.64-1.75) |
| High-potency cannabis | MI | **1.50 (1.23-1.82)** | 1.03 (0.82-1.29) | **1.56 (1.31-1.87)** | 1.12 (0.91-1.38) | 1.39 (0.88-2.20) | 0.82 (0.54-1.26) |
|  | CC | **1.48 (1.21-1.81)** | 1.03 (0.81-1.31) | **1.55 (1.28-1.86)** | 1.13 (0.90-1.40) | 1.34 (0.84-2.13) | 0.82 (0.52-1.29) |
|  | MI_10_ | **1.36 (1.10-1.69)** | 0.95 (0.83-1.10) | **1.47 (1.20-1.81)** | 1.10 (0.92-1.32) | 1.05 (0.68-1.64) | **0.70 (0.49-0.99)** |
|  | CC_10_ ^e^ | **1.40 (1.14-1.70)** | **1.37 (1.14-1.66)** | **1.50 (1.24-1.82)** | **1.49 (1.24-1.80)** | 1.11 (0.72-1.72) | 1.07 (0.70-1.63) |
| Unemployment | MI | **0.73 (0.58-0.92)** | 0.94 (0.77-1.14) | 0.79 (0.60-1.03) | 1.11 (0.92-1.33) | **0.48 (0.35-0.65)** | **0.48 (0.32-0.72)** |
|  | CC | **0.73 (0.58-0.92)** | 0.95 (0.78-1.16) | 0.79 (0.60-1.03) | 1.12 (0.93-1.35) | **0.48 (0.35-0.65)** | **0.49 (0.32-0.74)** |
|  | MI_10_ | 0.72 (0.50-1.03) | 0.83 (0.58-1.18) | 0.81 (0.52-1.25) | 1.20 (0.78-1.83) | **0.44 (0.28-0.69)** | **0.31 (0.13-0.72)** |
|  | CC_10_ ^e^ | **-** | **-** | **-** | **-** | - | - |
| Population density (people per km^2^) | MI | 1.04 (0.78-1.38) | 0.92 (0.77-1.11) | 1.12 (0.84-1.49) | 0.97 (0.82-1.16) | 0.78 (0.49-1.24) | 0.80 (0.53-1.20) |
|  | CC | 1.04 (0.78-1.38) | 0.92 (0.76-1.12) | 1.12 (0.84-1.49) | 0.97 (0.81-1.17) | 0.78 (0.49-1.24) | 0.80 (0.52-1.22) |
|  | MI_10_ | 0.95 (0.64-1.41) | 0.86 (0.67-1.11) | 1.12 (0.73-1.72) | 0.86 (0.63-1.16) | **0.50 (0.31-0.80)** | 0.81 (0.43-1.56) |
|  | CC_10_ ^e^ | **-** | **-** | **-** | **-** | - | - |

*FEP: first episode psychosis, IRR: incidence rate ratio, CI: confidence interval, MI/CC/MI_10_/CC_10_: see footnote b, below.*

*Bold: p<0.05 statistically significant*

*^a^z-standardized; IRR associated with 1 standard deviation changes.*

*^b^We report four analyses – our main results (MI) are replayed from Table 2, with three further sensitivity analyses: CC, MI_10_, CC_10_. These analyses are as follows:*

- *MI: Results from models using daily & high potency cannabis prevalence in controls in each setting following multiple imputation for missing cannabis data and post-stratification weighting for control representativeness*
- *CC: Results from models using daily & high potency cannabis prevalence in controls in each setting restricted to complete cannabis data on controls (see Supplement 2) and post-stratification weighting for control representativeness*
- *MI_10_: As MI, but restricted to 10 settings included in Di Forti et al (2019)*
- *CC_10_: Results from models using daily & high potency cannabis prevalence in controls in each setting restricted to complete cannabis data on controls, without post-stratification weighting, and restricted to 10 settings included in Di Forti et al (2019). The multivariable results in these rows replicate the correlational results for the prevalence of daily and high potency cannabis in controls and FEP incidence reported in Di Forti et al (2019) as closely as possible. One setting included in Di Forti et al (2019)’s original analysis was excluded here due to no data on ethnicity or migrant status being collected in that setting (Puy-de-Dome).*

*^c^Adjusted for age, sex, age-sex interaction, migrant/ethnic group, and all other variables in the table, except for CC_10_ (see footnote e)*

*^d^p-value=0.051*

*^e^Results reported for prevalence of daily and high-potency cannabis only, to replicate findings reported in Di Forti et al (2019). Multivariable results adjusted for age, sex and ethnicity only, but no other setting-level variables.*

*Supplemental Table 12ii: Prevalence of daily and high potency cannabis use based on complete case data without post-stratification weighting*

| **Catchment area** | **Controls**  **N (%)^1^** | **Missing daily cannabis use**  **N (%)^2^** | **Missing high potency cannabis use**  **N (%)^2^** | **% Daily cannabis (Complete data)^3^** | **% High-potency (Complete data)^3^** |
| --- | --- | --- | --- | --- | --- |
| Southeast London^d^ | 230 (17.2) | 7 (3.0) | 13 (5.7) | 11.7 | 26.7 |
| Cambridgeshire^d^ | 106 (7.9) | 0 (0.0) | 11 (10.4) | 3.8 | 9.7 |
| Amsterdam^d^ | 101 (7.6) | 1 (1.0) | 10 (10.0) | 12.9 | 56.3 |
| Gouda & Voorhout^d^ | 109 (8.2) | 1 (0.9) | 15 (13.8) | 5.5 | 19.5 |
| Madrid^d^ | 38 (2.8) | 0 (0.0) | 0 (0.0) | 10.5 | 15.8 |
| Barcelona^d^ | 37 (2.8) | 0 (0.0) | 1 (2.7) | 8.1 | 10.9 |
| Valencia | 32 (2.4) | 7 (21.9) | 9 (28.2) | 10.7 | 13.5 |
| Oviedo | 39 (2.9) | 9 (23.1) | 14 (35.9) | 5.3 | 9.7 |
| Santiago | 38 (2.8) | 5 (12.8) | 12 (31.6) | 0.0 | 0.0 |
| Cuenca | 38 (2.8) | 5 (12.8) | 5 (15.8) | 4.3 | 4.1 |
| Val-de-Marne^d^ | 100 (7.5) | 0 (0.0) | 7 (7.0) | 11.0 | 21.4 |
| Bologna^d^ | 65 (4.9) | 0 (0.0) | 8 (12.4) | 3.1 | 8.7 |
| Palermo^d^ | 100 (7.5) | 0 (0.0) | 4 (4.0) | 5.0 | 7.2 |
| Ribeirão Preto^d^ | 302 (22.6) | 3 (1.0) | 6 (2.0) | 6.7 | 1.7 |

*^a^Column percentages*

*^b^Row percentages (% controls with missing cannabis data per setting)*

*^c^Without post-stratification weighting*

*^d^Ten settings included by Di Forti et al (2019)*

**Supplement 13: Collaborators**

Silvia Amoretti, Barcelona Clinic Schizophrenia Unit, Hospital Clinic de Barcelona; Departament de Medicina, Institut de Neurociències (UBNeuro), Universitat de Barcelona (UB); Institut d’Investigacions Biomèdiques August Pi I Sunyer (IDIBAPS), CIBERSAM, ISCIII, Barcelona, Spain

Grégoire Baudin, Institut National de la Santé et de la Recherche Médicale, Créteil, France

Stephanie Beards, Department of Health Service and Population Research, Institute of Psychiatry, Psychology and Neuroscience, King's College London, London, the United Kingdom

Chiara Bonetto, Section of Psychiatry, Department of Neuroscience, Biomedicine and Movement, University of Verona, Verona, Italy

Angel Carracedo, Fundación Pública Galega de Medicina Xenómica, Hospital Clínico Universitario de Santiago de Compostela, Santiago de Compostela, Spain

Thomas Charpeaud, Fondation FondaMental, Créteil, France; and Université Clermont Auvergne, Clermont-Ferrand, France

Javier Costas, Fundación Pública Galega de Medicina Xenómica, Hospital Clínico Universitario de Santiago de Compostela, Santiago de Compostela, Spain

Doriana Cristofalo, Section of Psychiatry, Department of Neuroscience, Biomedicine and Movement, University of Verona, Verona, Italy

Pedro Cuadrado, Villa de Vallecas Mental Health Department, Villa de Vallecas Mental Health Centre, Hospital Universitario Infanta Leonor / Hospital Virgen de la Torre, Madrid, Spain

Daniella van Dam, Department of Psychiatry, Early Psychosis Section, Academic Medical Centre, University of Amsterdam, Amsterdam, the Netherlands

Aziz Ferchiou, Institut National de la Santé et de la Recherche Médicale, Créteil, France

Nathalie Franke, Department of Psychiatry, Early Psychosis Section, Academic Medical Centre, University of Amsterdam, Amsterdam, the Netherlands

Flora Frijda, Etablissement Public de Santé Maison Blanche, Paris, France

Enrique García Bernardo, Department of Psychiatry, Hospital General Universitario Gregorio Marañón, School of Medicine, Universidad Complutense, IiSGM, CIBERSAM, Madrid, Spain

Paz Garcia-Portilla, Department of Medicine, Psychiatry Area, School of Medicine, Universidad de Oviedo, Centro de Investigación Biomédica en Red de Salud Mental, Oviedo, Spain

Emiliano González, Department of Child and Adolescent Psychiatry, Institute of Psychiatry and Mental Health, Hospital General Universitario Gregorio Marañón, School of Medicine, Universidad Complutense, IiSGM, CIBERSAM, Madrid, Spain

Kathryn Hubbard, Department of Health Service and Population Research, Institute of Psychiatry, Psychology and Neuroscience, King's College London, London, the United Kingdom

Stéphane Jamain, Institut National de la Santé et de la Recherche Médicale, Créteil, France; and Faculté de Médecine, Université Paris-Est-Créteil, Créteil, France

Estela Jiménez-López, Department of Psychiatry, Servicio de Psiquiatría Hospital “Virgen de la Luz”, Cuenca, Spain

Marion Leboyer, Institut National de la Santé et de la Recherche Médicale, Créteil, France; and Faculté de Médecine, Université Paris-Est-Créteil, Créteil, France

Gonzalo López, Department of Child and Adolescent Psychiatry, Institute of Psychiatry and Mental Health, Hospital General Universitario Gregorio Marañón, School of Medicine, Universidad Complutense, IiSGM, CIBERSAM, Madrid, Spain

Esther Lorente-Rovira, Department of Psychiatry, School of Medicine, Universidad de Valencia, Centro de Investigación Biomédica en Red de Salud Mental (Centro de Investigación Biomédica en Red de Salud Mental), Valencia, Spain

Camila Marcelino Loureiro, Department of Neuroscience and Behavior, Division of Psychiatry, Ribeirão Preto Medical School, University of São Paulo, Ribeirão Preto, São Paulo, Brazil; and Center for Population Mental Health Research, University of São Paulo, São Paulo, Brazil

Giovanna Marrazzo, Unit of Psychiatry, “P. Giaccone” General Hospital, Palermo, Italy

Covadonga Martínez, Department of Child and Adolescent Psychiatry, Institute of Psychiatry and Mental Health, Hospital General Universitario Gregorio Marañón, School of Medicine, Universidad Complutense, IiSGM, CIBERSAM, Madrid, Spain

Mario Matteis, Department of Child and Adolescent Psychiatry, Institute of Psychiatry and Mental Health, Hospital General Universitario Gregorio Marañón, School of Medicine, Universidad Complutense, IiSGM, CIBERSAM, Madrid, Spain

Elles Messchaart, Rivierduinen Centre for Mental Health, Leiden, the Netherlands

Ma Soledad Olmeda, Department of Psychiatry, Hospital General Universitario Gregorio Marañón, School of Medicine, Universidad Complutense, IiSGM, CIBERSAM, Madrid, Spain

Mara Parellada, Department of Child and Adolescent Psychiatry, Institute of Psychiatry and Mental Health, Hospital General Universitario Gregorio Marañón, School of Medicine, Universidad Complutense, IiSGM, CIBERSAM, Madrid, Spain

Marta Rapado, Department of Child and Adolescent Psychiatry, Institute of Psychiatry and Mental Health, Hospital General Universitario Gregorio Marañón, School of Medicine, Universidad Complutense, IiSGM, CIBERSAM, Madrid, Spain

Ulrich Reininghaus, Department of Psychiatry and Neuropsychology, School for Mental Health and Neuroscience, Maastricht University Medical Centre, Maastricht, the Netherlands; and Department of Health Service and Population Research, Institute of Psychiatry, Psychology and Neuroscience, King's College London, London, the United Kingdom

Jean-Romain Richard, Institut National de la Santé et de la Recherche Médicale, Créteil, France

José Juan Rodríguez Solano, Puente de Vallecas Mental Health Department, Hospital Universitario Infanta Leonor / Hospital Virgen de la Torre, Centro de Salud Mental Puente de Vallecas, Madrid, Spain

Laura Roldán Diaz, Department of Child and Adolescent Psychiatry, Institute of Psychiatry and Mental Health, Hospital General Universitario Gregorio Marañón, School of Medicine, Universidad Complutense, IiSGM, CIBERSAM, Madrid, Spain

Mirella Ruggeri, Section of Psychiatry, Department of Neuroscience, Biomedicine and Movement, University of Verona, Verona, Italy

Emilio Sánchez, Department of Psychiatry, Hospital General Universitario Gregorio Marañón, School of Medicine, Universidad Complutense, IiSGM, CIBERSAM, Madrid, Spain

Crocettarachele Sartorio, Unit of Psychiatry, “P. Giaccone” General Hospital, Palermo, Italy

Franck Schürhoff, Institut National de la Santé et de la Recherche Médicale, Créteil, France; and Faculté de Médecine, Université Paris-Est-Créteil, Créteil, France; and Fondation FondaMental, Créteil, France

Fabio Seminerio, Department of Biomedicine, Neuroscience, and Advanced Diagnostics, Section of Psychiatry, University of Palermo, Palermo, Italy

Rosana Shuhama, Department of Neuroscience and Behavior, Division of Psychiatry, Ribeirão Preto Medical School, University of São Paulo, Ribeirão Preto, São Paulo, Brazil; and Center for Population Mental Health Research, University of São Paulo, São Paulo, Brazil

Lucia Sideli, Department of Biomedicine, Neuroscience, and Advanced Diagnostics, Section of Psychiatry, University of Palermo, Palermo, Italy

Simona A. Stilo, Department of Health Service and Population Research, Institute of Psychiatry, Psychology and Neuroscience, King's College London, London, the United Kingdom; and Department of Psychosis Studies, Institute of Psychiatry, Psychology and Neuroscience, King's College London, London, the United Kingdom

Federico Suprani, BolognaTranscultural Psychosomatic Tream (BoTPT), Department of Medical and Surgical Sciences, Bologna University, Bologna, Italy

Andrei Szöke, Université Paris-Est-Créteil (UPEC), AP-HP, Hôpitaux Universitaires « H. Mondor », DMU IMPACT, INSERM, IMRB, Translational Neuropsychiatry, Fondation FondaMental, F-94010 Créteil, France

Fabian Termorshuizen, Department of Psychiatry and Neuropsychology, School for Mental Health and Neuroscience, Maastricht University Medical Centre, Maastricht, the Netherlands

Sarah Tosato, Section of Psychiatry, Department of Neuroscience, Biomedicine and Movement, University of Verona, Verona, Italy

Anne-Marie Tronche, Fondation FondaMental, Créteil, France; and CMP B CHU, Clermont-Ferrand, France; and Université Clermont Auvergne, Clermont-Ferrand, France

Els van der Ven, Department of Psychiatry and Neuropsychology, School for Mental Health and Neuroscience, Maastricht University Medical Centre, Maastricht, the Netherlands; and Rivierduinen Centre for Mental Health, Leiden, the Netherlands

**Supplement 14: Supplemental References**

Di Forti M, Quattrone D, Freeman TP, et al. The contribution of cannabis use to variation in the incidence of psychotic disorder across Europe (EU-GEI): a multicentre case-control study. *Lancet Psychiatry.* 2019;6(5):427-436.

European Monitoring Centre for Drugs and Drug Addiction, Spanish Ministry of Health and Consumer Affairs. *Spain national report (2011 Data) to the EMCDDA 2012.* Madrid: Government Delegation for National Plan on Drugs; 2012.

European Monitoring Centre for Drugs and Drug Addiction. *European drug report 2016: trends and developments.* Luxembourg: Publications Office of the European Union; 2016.

Gayer-Anderson C, Jongsma HE, Di Forti M, et al. The EUropean Network of National Schizophrenia Networks Studying Gene–Environment Interactions (EU-GEI): incidence and first-episode case–control programme. Soc Psychiatry *Psychiatr Epidemiol*. 2020;55(5):645-657.

Hardwick S, King L. *Home Office cannabis potency study 2008*. Sandridge, St Albans: Home Office Scientific Development Branch Sandridge; 2008.

Hilbe JM. *Negative Binomial Regression.* 2nd ed. Cambridge: Cambridge University Press; 2011.

March D, Hatch SL, Morgan C, et al. Psychosis and place. *Epidemiol Rev.* 2008;30: 84-100.

Jongsma HE, Gayer-Anderson C, Lasalvia A, et al. Treated incidence of psychotic disorders in the multinational EU-GEI study. *JAMA Psychiatry.* 2018;75(1):36-46.

Lopes de Oliveira G, Voloch MH, Sztulman GB, Negrini Neto O, Yonamine M. Cannabinoid contents in cannabis products seized in São Paulo, Brazil, 2006-2007. *Forensic Toxicol.* 2008;26(1):31-35.

Niesink R, Rigter S. *THC-concentraties in wiet, nederwiet en hasj in Nederlandse coffeeshops (2012–2013).* Utrecht: Trimbos-instituut, 2013.

Niesink RJM, Rigter S, Koeter MW, Brunt TM. Potency trends of Δ9-tetrahydrocannabinol, cannabidiol and cannabinol in cannabis in the Netherlands: 2005-15. *Addiction.* 2015;110(12):1941-1950.

Observatoire Français des Drogues et des Toxicomanies (ODFT). *6th edition of Drugs, key data (June 2015).* La Plaine Saint-Denis; 2015.

Potter DJ, Clark P, Brown MB. Potency of Δ9-THC and other cannabinoids in cannabis in England in 2005: Implications for psychoactivity and pharmacology. *J Forensic Sci.* 2008;53(1):90-94.

Rucker J, Newman S, Gray J, et al. OPCRIT+: an electronic system for psychiatric diagnosis and data collection in clinical and research settings. *Br J Psychiatry.* 2011;199(2):151-155.

Valliant, R., & Dever, J. A. (2018). Survey weights: a step-by-step guide to calculation. Stata Press.

Zamengo L, Frison G, Bettin C, Sciarrone R. Cannabis potency in the Venice area (Italy): Update 2013. *Drug Test Anal.* 2015;7(3):255-258.
